# Phase 1b Study of Dazostinag plus Pembrolizumab after Hypofractionated Radiotherapy in Patients with Select Advanced Solid Tumors

**DOI:** 10.1158/2767-9764.CRC-25-0566

**Published:** 2025-12-31

**Authors:** Benjamin T. Cooper, Wade T. Iams, David B. Page, Yuan Yuan, Naamit K. Gerber, Jason J. Luke, John P. Gibbs, Richard C. Gregory, Kwok-Kin Wong, Jiehui Deng, Samanthi A. Perera, Kai Ding, Emily R. Roberts, Allison Berger, Camilla L. Christensen, Erica Xin Tong, Angel E. Maldonado López, Vicky A. Appleman, E. Jane Leonard, Alexander Parent, Yu-Chung Huang, Camden Bay, Cong Li, Neil Lineberry, Jeffrey Raizer, Daniel J. Olson, Steven J. Chmura

**Affiliations:** 1 Radiation Oncology, NYU Langone School of Medicine, New York, New York.; 2 Oncology, Vanderbilt University, Nashville, Tennessee.; 3 Medicine, Earle A. Chiles Research Institute, Providence Cancer Institute, Portland, Oregon.; 4 Medical Oncology, Cedars Sinai Medical Center, Los Angeles, California.; 5 Hematology/Oncology, UPMC Hillman Cancer Center, University of Pittsburgh, Pittsburgh, Pennsylvania.; 6 Quantitative Clinical Pharmacology, Takeda Development Center Americas, Inc. (TDCA), Cambridge, Massachusetts.; 7 Oncology Precision and Translational Medicine, Takeda Development Center Americas, Inc. (TDCA), Cambridge, Massachusetts.; 8 Department of Medicine, Laura & Issac Perlmutter Cancer Center, NYU Langone Health, New York, New York.; 9 Oncology Drug Discovery Unit, Takeda Development Center Americas, Inc. (TDCA), Cambridge, Massachusetts.; 10 Statistical and Quantitative Sciences, Takeda Development Center Americas, Inc. (TDCA), Cambridge, Massachusetts.; 11 Research and Development, Takeda Development Center Americas, Inc. (TDCA), Cambridge, Massachusetts.; 12 Oncology, Takeda Development Center Americas, Inc. (TDCA), Cambridge, Massachusetts.; 13 Medicine, University of Chicago, Chicago, Illinois.; 14 Radiation and Cellular Oncology, University of Chicago, Chicago, Illinois.

## Abstract

**Purpose:**

We present the preclinical rationale and clinical data from a phase 1b trial investigating the STING agonist dazostinag plus pembrolizumab following hypofractionated radiotherapy (RT) in patients with advanced non–small cell lung cancer (NSCLC), triple-negative breast cancer (TNBC), or squamous cell carcinoma of the head and neck (SCCHN) whose disease had progressed on prior checkpoint inhibitors (CPI; NCT04879849).

**Patients and Methods:**

Eligible patients received radiation (8 Gy × 3 fractions) followed (≥40 hours) by pembrolizumab 200 mg every 3 weeks and dazostinag in escalating doses (0.2–5.0 mg). Primary endpoints were safety and tolerability. Secondary endpoints included preliminary antitumor activity in irradiated and nonirradiated lesions, pharmacokinetic analyses, and pharmacodynamic analyses.

**Results:**

Preclinical studies demonstrated tumor control and enhanced intratumoral immune activation in mice treated with dazostinag plus radiation. Thirty-four patients (NSCLC: 15, SCCHN: 10, and TNBC: 9) with a median number of six prior treatments were enrolled. Thirty-three (97.1%) patients reported treatment-emergent adverse events (TEAE), none were dose-limiting toxicities; the most common were fatigue (52.9%), constipation (26.5%), and cough (20.6%). Dazostinag-related TEAEs occurred in 17 patients (50.0%); the most common were fatigue (26.5%), chills (8.8%), diarrhea, arthralgia, and myalgia (5.9% each). Antitumor activity, per RECIST v.1.1, was confirmed in two (7.1%) patients (one complete response and one partial response). Pharmacodynamic analyses indicated activation of STING and IFNγ pathways across multiple dose levels and induced immune responses, consistent with preclinical studies.

**Conclusions:**

Dazostinag, combined with pembrolizumab after RT, was well tolerated and demonstrated clinical activity in some patients with advanced/metastatic tumors whose disease had progressed on CPIs.

**Significance:**

Dazostinag, an intravenous STING agonist, combined with radiation, demonstrated tumor control and enhanced intratumoral immune activation, preclinically. In phase 1b, dazostinag plus pembrolizumab following RT had a manageable safety profile and provided clinical benefit for some heavily pretreated patients with advanced/metastatic solid tumors whose disease had progressed on CPIs.

## Introduction

Checkpoint inhibitors (CPI) have revolutionized cancer treatment, particularly in tumors considered immunogenic, but resistance to CPIs remains a challenge (1–4). Many patients with advanced cancer do not respond to CPIs due to primary resistance or relapse after a period of tumor control due to acquired or secondary resistance (1–4). Impaired IFN signaling, immune escape, altered antigen presentation, and immunosuppressive tumor phenotypes have been described as CPI resistance mechanisms (5, 6). Stimulating innate immune cells to create a proinflammatory tumor environment and activate type I IFN (IFN-I) signaling may enhance adaptive antitumor immunity, helping to overcome CPI resistance (7–9). Advances in understanding the tumor microenvironment (TME) and development of novel therapeutics to target the TME provide potential for combining therapies with complementary mechanisms of action to overcome resistance to CPIs and enhance antitumor immunity (3).

Radiotherapy (RT) has a direct cytotoxic impact on tumor cells by inducing DNA damage (10, 11). The resulting cell death generates cytosolic DNA and releases tumor antigens extracellularly, leading to activation of immune cells (including tumor-specific T cells) that can infiltrate distant cancerous lesions and enable their regression. This systemic antitumor immune response, termed the “abscopal effect” (10–12), is rarely observed with radiation alone in the clinical setting (11, 12). Conversely, RT can also result in immunosuppression, for example via induction of PD-L1 expression (13). Several studies have investigated the combined effect of RT and CPIs in solid tumors, with overall response rates (ORR) ranging from 13.2% to 45.9% (14–19), and potential evidence of the abscopal effect (keeping in mind the impact of the CPI), with ORRs in nonirradiated lesions ranging from 10% to 29%; refs. 15–17, 20–22). However, many challenges remain in the development of combination CPI/RT treatment. These include determining the optimal agents for combination, treatment sequencing and timing, drug and radiation doses, the number of lesions to irradiate, how to determine the benefit of CPI versus the abscopal effect with CPI/RT, as well as how to define and measure activity in irradiated versus nonirradiated lesions (11, 12).

In preclinical mouse models, radiation-induced cell death was shown to result in the activation of the cyclic guanosine monophosphate–adenosine monophosphate synthase STimulator of INterferon Genes (STING) pathway, which led to a STING-dependent IFN-I pathway induction (13, 23). The STING pathway is known to mediate innate immunity via the induction and production of IFN-I and other inflammatory cytokines (24, 25). Preclinical studies have also suggested that combining a CPI and a STING agonist enhances antitumor activity, including in tumors unresponsive to PD-1 inhibition alone (26).

Dazostinag (TAK-676) is a novel synthetic intravenously delivered STING agonist that activates the innate immune system and mobilizes adaptive immunity (27, 28). In preclinical studies, dazostinag promoted tumor control with observed innate and adaptive immune responses via activation of dendritic, NK, and CD8^+^ T cells (27). In the first-in-human phase 1 dose escalation study (iintune-1, NCT04420884), dazostinag as a single agent and in combination with pembrolizumab was well tolerated and demonstrated preliminary antitumor activity in patients with advanced/metastatic solid tumors (29).

The combination of STING agonism, hypofractionated radiation, and CPIs is hypothesized to synergistically enhance antitumor immunity by targeting complementary mechanisms. In this study, we present the supportive preclinical experiments and the clinical data from a phase 1 dose escalation trial designed to investigate the safety, preliminary antitumor activity, and pharmacodynamic impact of dazostinag in combination with pembrolizumab following hypofractionated RT in patients with advanced/metastatic non–small cell lung cancer (NSCLC), triple-negative breast cancer (TNBC), or squamous cell carcinoma of the head and neck (SCCHN) who had progressed on prior CPI therapy (NCT04879849).

## Patients and Methods

### Preclinical studies

#### Mouse models and cell lines

All procedures carried out in these experiments were conducted at Covance in compliance with the applicable laws, regulations, and guidelines of the NIH and with the approval of Covance’s Animal Care and Use Committee. Covance is an American Association for Accreditation of Laboratory Animal Care (AAALAC)-accredited facility. Female BALB/c mice (BALB/cAnNHsd; Envigo) were fed a Teklad 291815 rodent diet (supplied by Envigo Bioproducts, Inc.) and fed water *ad libitum*. The mice were inoculated with 1.0 × 10^6^ EMT6 cells in the mammary fat pad and allowed to establish for 8 or 9 days before initiation of treatment as described below. Implantation of tumor cells was performed at 7 to 8 weeks of age, and hydrogel was provided at body weight losses >10%.

EMT6 cells used in the experiments were obtained as frozen stocks from the ATCC (CRL-2755; RRID: CVCL-1923), cultured in DMEM supplemented with 10% non–heat-inactivated FBS and 1% penicillin/streptomycin/L-Glutamine, and expanded. Short tandem repeat profiling was performed for cell line identification, and cells were not cultured for more than 6 months following resuscitation. During culture time, cells were fed and split as needed to avoid over confluence and achieve the required cell counts. Cell dissociation for passaging and implantation was performed by incubating with 0.25% trypsin/2.21 mmol/L EDTA in Hank’s balanced salt solution for 5 to 15 minutes at 37°C. Cells were used for experiments as needed during the culture period but never exceeding passage 10. Cells were tested for *Mycoplasma* sp. and murine pathogen via PCR. For implantation, mice were lightly anesthetized via inhalation of isoflurane/oxygen and an inoculum of 1.0 × 10^6^ trypan blue–excluding cells suspended in 50 μL of DMEM was orthotopically injected into the right mammary fat pad (pad #4).

#### *In vivo* Small Animal Radiation Research Platform irradiation

An Xstrahl Life Sciences Small Animal Radiation Research Platform, which allows for highly targeted irradiation (mimicking that applied to human patients), was used to treat mice in a supine position for best access to the irradiation site. Treatment planning was performed with MuriPlan software (Xstrahl, Inc.) and open-field imaging with a 60 kV and 0.5 mA system. Treatment was performed with 220 kV, 13.0 mA using a 10 mm × 10 mm collimator with a dose rate of 2.42 Gy/minute. Animals were imaged and treated under 1% to 2% isoflurane anesthesia. For repeated treatments, the same treatment plan was applied and adjusted for changes in animal positioning or target alteration over time.

#### Treatment for efficacy study

All mice were sorted into study groups based on caliper estimation of tumor burden. The mice were distributed to ensure that the mean tumor burden for all groups was within 10% of the overall mean tumor burden of the study population (Supplementary Table S1). Radiation or mock radiation (similar animal handling and procedure with no actual radiation delivery) was given on three consecutive days (days −4 to −2). Dazostinag (1.0 mg/kg) and/or αPD-1 antibody (clone RMP1-14, Bio X Cell, cat. #BP0146; RRID: AB_10949053; 10.0 mg/kg) were respectively administered intravenously and intraperitoneally on days 0, 3, and 5 (every 3 days for three doses). As controls for dazostinag and αPD-1, mice received PBS or an isotype-matched control antibody using the same route of administration and schedule, respectively (Supplementary Fig. S1A). Tumor growth was monitored until a humane endpoint (tumor burden of >2,000 mm^3^) was reached.

#### Rechallenge of tumor-free survivors

Some mice from groups 6 (1.0 mg/kg dazostinag, 10.0 mg/kg isotype control, and 8 Gy radiation) and 8 (1.0 mg/kg dazostinag, 10.0 mg/kg anti–PD-1, and 8 Gy radiation) survived and demonstrated full tumor regression (previously observable tumors were no longer measurable after treatment). These surviving, tumor-free mice were observed for an extended period of time, after which they (along with age-matched controls, group 10, no treatment and no radiation) were reinoculated via subcutaneous administration in the right flank (pad #9) with 1.0 × 10^6^ trypan blue–excluding EMT6 cells resuspended in 50 μL of DMEM. Tumor growth following this new inoculation was measured in the absence of treatment to evaluate immune memory.

#### Treatment for pharmacodynamic evaluation

In a separate experiment, EMT6 tumor-bearing mice received focal radiation (8 Gy) for three consecutive days (days −4 to −2 post inoculation) and/or intravenous dazostinag (1.0 mg/kg) on day 0 for single-dose groups or days 3 and 5 for double-dose groups (Supplementary Fig. S1A). All mice were sorted into study groups based on caliper estimation of tumor burden. The mice were distributed to ensure that the mean tumor burden for all groups was within 10% of the overall mean tumor burden for the study population (Supplementary Table S2).

#### Sample collection

Mice were euthanized, and the levels of cytokines were assessed by Meso Scale Discovery. Multiplex cytokine assays at 24 hours after the last mock/radiation treatment (groups 1 and 4), 24 and 120 hours after control (PBS)/dazostinag dose for the single-dose groups (groups 2, 5, 7, and 9) and 48 hours after the second dose of control (PBS)/dazostinag for the double-dose groups (groups 3, 6, 8, and 10). Fresh whole blood was collected via cardiac puncture into a dipotassium EDTA (K_2_ EDTA) tube, and fresh lymph nodes were collected by excision and placed into tubes containing cold PBS. All samples were stored on wet ice and processed for flow cytometry analysis. Plasma from fresh whole blood collected via cardiac puncture into a K_2_ EDTA tube was prepared and frozen at −80°C until cytokine analysis. A portion of the bisected tumors was excised, placed in 10% neutral-buffered formalin, and embedded into paraffin. The remaining tumor was snap-frozen in liquid nitrogen to be processed for cytokine analysis.

#### Cytokine induction

Cytokine pharmacodynamics were measured using an electrochemiluminescence-based assay by Meso Scale Discovery, which measured IFNγ-induced protein-10 (IP-10), IL16, IL17 A/F, IL27p28/IL30, IL33, IL8, monocyte chemoattractant protein-1 (MCP-1), macrophage inflammatory protein (MIP)-1α, MIP-2, IFNγ, IL10, IL1β, IL2, IL4, IL5, IL6, keratinocyte chemoattractant/human growth-regulated oncogene, and TNFα according to manufacturer’s instructions. Briefly, 300 μg of tumor lysate, or either 2.5 or 10 μL of plasma in a total volume of 50 μL, were added to Meso Scale Discovery plates with appropriate capture antibodies and incubated at room temperature for 1 hour. Plates were washed with buffer [2% Tween 20 in PBS (PBST)] and then incubated with Sulfo-Tag–conjugated detection antibody for 1 hour at room temperature. After washing with PBST, reading buffer (Meso Scale Discovery) was added and signal was measured using MESO QuickPlex SQ 120MM instrument (Meso Scale Discovery). Data were analyzed using Discovery Workbench v4, transferred to Microsoft Excel for organization, and plotted using GraphPad Prism v9.

#### Multiplex immunofluorescence

Paraffin-embedded EMT6 tumors from control and treated groups were stained in a Bond RX (Leica Biosystems) with a proprietary T-cell panel.

#### *In vivo* immune cell activation and proliferation

Immunophenotyping was performed on the draining lymph nodes of tumor-bearing mice treated with radiation and dazostinag using two different validated proprietary antibody panels. Infiltration of immune cells was assessed by flow cytometry at the same time points as described above for cytokine analysis.

#### Flow cytometry

Fresh whole blood and freshly excised lymph nodes were prepared and stained by contract research organization Covance using two different antibody panels, a T-cell panel [CD45, CD4, CD223 (LAG-3), CD366 (Tim-3), CD62L, CD25, CD278 (ICOS), CD279 (PD-1), CD49b, CD335, CD8, CD3, CD44, CD69, granzyme B, FoxP3, and Ki67] and a myeloid-derived suppressor cell panel (CD45, CD19, CD11b, Ly-6C, Ly-6G, and F4/80).

### Phase 1 study design and treatment

This open-label, single-arm, dose-escalation, United States–based multicenter phase 1 study recruited patients with advanced or metastatic NSCLC, TNBC, or SCCHN whose disease had progressed on or after CPIs in a prior line of therapy (Supplementary Fig. S1B). This population represents three solid tumor types for which pembrolizumab is an approved therapy [KEYTRUDA (pembrolizumab) US FDA prescribing information. Accessed November 11, 2025. Available at https://www.merck.com/product/usa/pi_circulars/k/keytruda/keytruda_pi.pdf]. Eligible patients received three consecutive fractions (8 Gy) of image-guided radiation treatment between day −8 and day −1. A minimum of 40 hours from the last RT was then required before administration of intravenous pembrolizumab 200 mg on day 1 plus escalating doses (0.2–5.0 mg) of systemic intravenous dazostinag on days 1 (1 hour after pembrolizumab), 8, and 15 of a 21-day cycle. Dazostinag dose escalation was guided by Bayesian Optimal Interval statistical design. The range of dazostinag dose levels (0.2–5.0 mg) in combination with 200 mg pembrolizumab was associated with an acceptable safety profile in the iintune-1 study (30).

### Patients

Eligible patients were ≥18 years old with confirmed advanced/metastatic NSCLC, TNBC, or SCCHN who had progressed on CPI therapy. Patients must have had at least two measurable lesions (per RECIST v.1.1). At least one and up to three target lesions could be irradiated, with ≥1 target lesion remaining nonirradiated. The representativeness of the study population is described in Supplementary Table S3, and full eligibility criteria are in the Supplementary Methods.

### Endpoints and assessments

The primary endpoints were the frequency and severity of treatment-emergent adverse events (TEAE), the number of patients with dose-limiting toxicities (DLT, see definition in the Supplementary Methods), rates of serious TEAEs, and rates of TEAEs leading to dose modification or treatment discontinuation. Safety and toxicity were evaluated using the NCI Common Terminology Criteria for Adverse Events v5.0. TEAEs and serious TEAEs were reported from the date of signing the informed consent through 30 days after the last dose of study treatment or start of subsequent treatment, which ever occurred first. When considered immune-mediated by the investigator, TEAEs were monitored through 90 days after the last dose of study treatment/start of subsequent treatment. Secondary endpoints included the ORR and duration of response (DOR) for overall, irradiated, and nonirradiated tumor lesions. Disease status and treatment response were based on local investigator assessment according to RECIST v.1.1 using CT or MRI scans with intravenous contrast at screening (within 28 days prior to the first study radiation treatment), once at the end of cycle two (day 15–22), then at the end of every three cycles (days 15–21) for the first year, and then at the end of every six cycles (plus 7 days) thereafter until progressive disease (PD) or the start of subsequent treatment. The same lesions identified as target and nontarget at screening were followed at each subsequent response assessment. Response to treatment was also assessed using a modified version of itRECIST (31), overall, in irradiated and nonirradiated lesions. Baseline lesions were categorized as target irradiated (T-I), target, nonirradiated (T-NI), nontarget irradiated (NT-I), and nontarget nonirradiated (NT-NI) according to an algorithm. The same target and nontarget lesions selected as part of the RECIST v.1.1 assessment at screening may have been used for the nonirradiated target or nontarget lesions selected under the modified itRECIST assessment. One and up to three lesions may have been irradiated. At least one lesion must have been T-I, whereas the remaining two may have been T-I or NT-I. One to three measurable lesions were designated as T-I and were used to evaluate the irradiated lesion response. One to five measurable lesions were designated as T-NI. The irradiated response was based entirely on T-I lesions aggregating the sum of the largest diameter for all of the irradiated target lesions, whereas the nonirradiated response was based entirely on T-NI lesions aggregating the sum of the largest diameter for all of the nonirradiated target lesions. Overall target lesion response, nontarget lesion response, and new lesion appearance were defined as per RECIST v.1.1 and combined similarly to determine the itRECIST overall response for each visit. The overall response included all lesions classified as target at baseline (sum of diameters of T-I and T-NI) combined vs. the sum of diameters at nadir, all nontarget lesions (NT-I and NT-NI) combined (classified as absent, present, or collectively showing unequivocal progression), and new lesions.

Exploratory endpoints included the pharmacokinetic (PK) assessment of dazostinag plasma concentrations over time and pharmacodynamic analyses of changes in STING gene signature, as well as changes in cytokines and immune cell subsets following treatment. Blood samples were collected before infusion (0 hours), at the end of infusion (EoI), and 3 and 6 hours after dazostinag infusion on cycle 1 day 1 (C1D1) and C1D15 for PK evaluation of dazostinag plasma concentrations using a LC/MS-MS method. Plasma and blood pharmacodynamic samples were collected at screening, before infusion (0 hours), and 6 hours after dazostinag infusion on C1D1. Induction of STING pathway activation was assessed in peripheral blood by calculating a STING gene signature score (Q2 Expression Analysis and Qiagen) derived from whole-transcriptome RNA sequencing (RNA-seq) data of peripheral blood mononuclear cells from healthy donors treated *ex vivo* with dazostinag. For cytokine and chemokine detection, Meso Scale Discovery V-Plex and V-Plex plus proinflammatory (cat. #K151QR/K15049/K15349) and chemokine (cat. #K15047/K15055/K156NO) panel multiplex kits were used to perform assays on collected plasma samples according to the manufacturer’s instructions (BioAgilytix). These assays used the Meso Scale Discovery S600 instruments for detection and Gen5 Secure software for data capture. STING signature and cytokine fold change levels were normalized to C1 0 hour (C1H0). Single-cell RNA-seq (scRNA-seq) was performed on pre- and on-treatment tumor samples from one patient to assess changes in immune cells following treatment with dazostinag and pembrolizumab after RT. The paired biopsied lesions (that were not planned to be irradiated) were collected at screening and after treatment with RT, dazostinag, and pembrolizumab at C1D15. Data visualization was performed using Uniform Manifold Approximation and Projection for Dimension Reduction (UMAP; ref. 32).

scRNA-seq was performed using Cell Ranger Single-Cell Software Suite for sample demultiplexing, mapping (GRCm38 genome reference), barcode processing, and gene expression quantification. Filtered gene–barcode matrices that contained only barcodes with unique molecular identifier counts that passed the threshold for cell detection were imported into R as a Seurat v5 (33) object for further analysis. Cells with fewer than 200 genes detected or greater than 10% mitochondrial RNA content were deemed as low quality and excluded from analysis. RNA counts were normalized using *Seurat::SCTransform* function with regressions of the cell-cycle score and ribosomal and mitochondrial percentages for each sample. Multiple samples were integrated using Seurat standard scRNA-seq integration workflow with 3,000 anchor genes, followed by cell clustering using Leiden algorithm and visualization using UMAP (32). Multiple resolutions were tested for optimal cell type identifications. Cell types were manually annotated based on canonical cell type markers, and differentially expressed genes of each cluster were identified using *Seurat::FindAllMarkers* function with a logistic regression model.

### Statistical analysis

Study populations are described in full in the Supplementary Methods. The study planned to enroll 46 patients (to achieve a maximum of 39 DLT-evaluable patients for the dose escalation), with ≥3 patients per cohort. Patient demographics, baseline characteristics, and primary endpoints (safety) were summarized descriptively. ORRs were summarized using descriptive statistics with 95% exact confidence intervals for RECIST v.1.1 and modified itRECIST (overall, irradiated, and nonirradiated), whereas the DOR was analyzed using the Kaplan–Meier method.

### Ethical statement

All preclinical procedures were conducted in compliance with the applicable laws, regulations, and guidelines of the NIH and with the approval of Covance’s Animal Care and Use Committee, in line with the AAALAC. Study reports were drafted by Covance MILL202004R2C3 (MI4325), MILL202015R0C1 (MI4509), and MILL202005R4 (MI4764).

The clinical study was conducted according to the Declaration of Helsinki and the Harmonized Tripartite Guideline for Good Clinical Practice from the International Council for Harmonization of Technical Requirements for Pharmaceuticals for Human Use. All patients provided written informed consent. Each investigator conducted the study according to applicable local regulatory requirements and following approvals of local institutional review boards (IRB) and independent ethics committees: IRB #S20-01768 (NYU Langone School of Medicine; principal investigator, B.T. Cooper), IRB #210129 (Vanderbilt University; principal investigator, W.T. Iams), Advarra central IRB #SSU00157360 (Earle A. Chiles Research Institute, Providence Cancer Institute; principal investigator, D.B. Page), IRB #STUDY00001378 and IRB ID CR00004504 (Cedars Sinai Medical Center; principal investigator, Y. Yuan), IRB #STUDY21060024 (UPMC Hillman Cancer Center, University of Pittsburgh; principal investigator, J. Luke), and IRB #IRB20-2093-CR004 (University of Chicago; principal investigator, D.J. Olson). No steering committee or data safety monitoring committee were used in this study, but safety was monitored by the dazostinag safety monitoring team. This clinical trial is registered on Clinicaltrials.gov (NCT04879849) and World Health Organization (U1111-1252-0338) databases.

## Results

### Preclinical results

**Figure 1.**
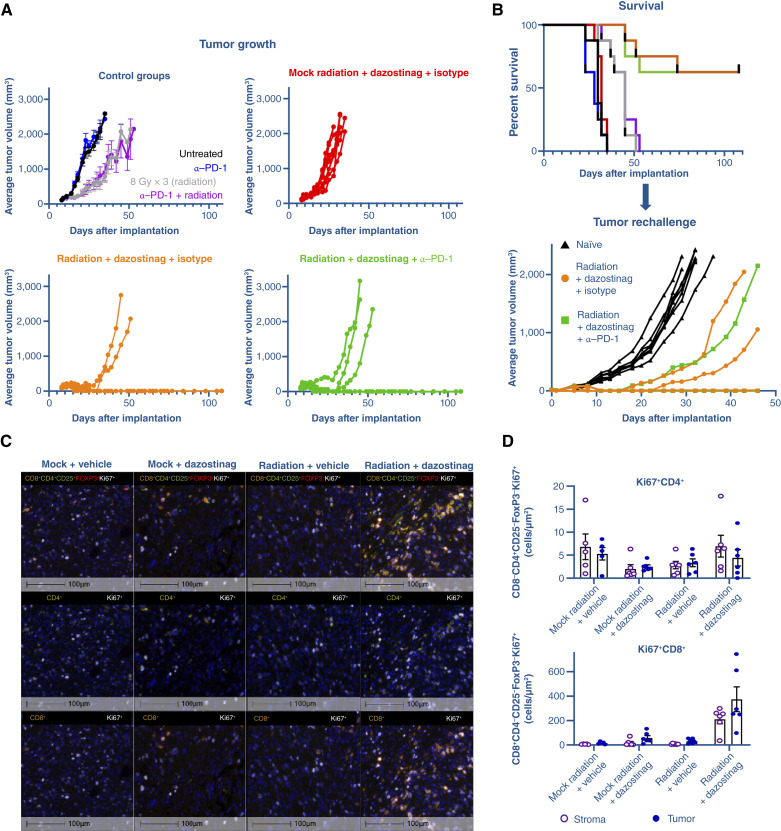
**A** and **B,** Preclinical antitumor activity and (**C** and **D**) analysis of tumor immune cells in EMT6 tumor-bearing mice treated with dazostinag following fractionated radiation. α, anti; Gy, gray; mock, mock radiation treatment.

EMT6 tumor-bearing mice were treated with dazostinag, fractionated radiation, or both dazostinag and fractionated radiation. Dazostinag combined with fractionated radiation led to durable tumor regression in *n* = 6/8 mice, whereas dazostinag or radiation treatment alone did not result in tumor regression. The addition of an antibody against mouse PD-1 (anti–mPD-1) did not further enhance the efficacy of dazostinag plus fractionated radiation regimen, with *n* = 5/8 regressions (Fig. 1A). All treatments were well tolerated (no mice suffered loss of >20% body mass). To establish whether mice experiencing tumor regression had developed immune memory, tumor growth was measured after tumor rechallenge in the opposite flank in the absence of treatment. Tumor growth was observed in *n* = 8/8 age-matched controls; only in *n* = 2/5 mice that had previously received radiation, dazostinag, and isotype; and only in *n* = 1/5 mice previously treated with radiation and dazostinag plus anti–mPD-1. These results demonstrated generation of immune-mediated memory against EMT6 (Fig. 1B).

Elevated levels of peripheral cytokines CXC ligand-10 (also called IP-10), TNFα, IL6, MIP-1α, and IFNγ were observed in plasma from EMT6 tumor-bearing mice 24 hours after receiving a single dose of dazostinag, with or without preceding radiation, and the cytokine levels returned to baseline within 120 hours. In tumor samples, only mice treated with both dazostinag and radiation had increased levels of IFNγ on day 5, suggestive of an adaptive immune response with the combination regimen (Supplementary Fig. S2A). Analysis of tumor-draining lymph nodes indicated that CD4^+^ T cells were largely unaffected by radiation and dazostinag and that dazostinag alone could induce a higher number of CD8^+^ T cells at day 5. Increases in NK cells and activated CD8^+^ T cells (CD69^+^ or granzyme B+) were observed following radiation treatment and were similar with or without dazostinag. A reduction in naïve CD8^+^ T cells was also reported following radiation treatment, consistent with activation of this subset (Supplementary Fig. S2B). Enhanced proliferation of CD8^+^ T cells in tumor was observed in response to radiation combined with dazostinag when compared with dazostinag or radiation used alone, demonstrating the potential of this combination for enhanced T-cell response (Fig. 1C and D).

### Phase 1 clinical study

At data cutoff of July 3, 2024, 34 patients had been enrolled across six sites in the United States. Patient baseline demographics and disease characteristics are shown in Table 1. The median age was 61 years, 19 patients were male (55.9%), and 24 patients were White (70.6%). Fifteen patients had NSCLC, 10 had SCCHN, and nine had TNBC. *KRAS* mutation was detected in three patients with NSCLC, whereas of the nine patients with SCCHN and available p16 results, six were positive for p16 expression by IHC, which is associated with human papillomavirus.

**Table 1.** Patient demographics and disease characteristics at baseline (safety population).

*n* (%), unless otherwise stated	Radiation only^a^ *n* = 2	Hypofractionated RT + dazostinag + pembrolizumab							Overall *N* = 34
		Dazostinag 0.2 mg *n* = 4	Dazostinag 0.4 mg *n* = 2	Dazostinag 0.8 mg *n* = 4	Dazostinag 1.6 mg *n* = 3	Dazostinag 2.5 mg *n* = 7	Dazostinag 3.5 mg *n* = 6	Dazostinag 5.0 mg *n* = 6	
Median age, years (range)	73.0 (68–78)	49.0 (38–65)	65.5 (61–70)	56.0 (43–65)	61.0 (48–70)	67.0 (38–91)	64.0 (40–75)	54.5 (41–69)	61.0 (38–91)
Male	2 (100)	2 (50.0)	2 (100)	3 (75.0)	1 (33.3)	4 (57.1)	2 (33.3)	3 (50.0)	19 (55.9)
Types of cancers	​	​	​	​	​	​	​	​	​
NSCLC	1 (50.0)	2 (50.0)	1 (50.0)	1 (25.0)	2 (66.7)	3 (42.9)	3 (50.0)	2 (33.3)	15 (44.1)
TNBC	0	1 (25.0)	0	1 (25.0)	1 (33.3)	1 (14.3)	2 (33.3)	3 (50.0)	9 (26.5)
SCCHN	1 (50.0)	1 (25.0)	1 (50.0)	2 (50.0)	0	3 (42.9)	1 (16.7)	1 (16.7)	10 (29.4)
NSCLC – TPS/CPS	​	​	​	​	​	​	​	​	​
<1%	1 (50.0)	1 (25.0)	0	1 (25.0)	1 (33.3)	0	0	1 (16.7)	5 (14.7)
1%–49%	0	0	1 (50.0)	0	0	1 (14.3)	1 (16.7)	0	3 (8.8)
≥50%	0	1 (25.0)	0	0	1 (33.3)	0	1 (16.7)	1 (16.7)	4 (11.8)
No PD-L1 test/unknown	0	0	0	0	0	2 (28.6)	1 (16.7)	0	3 (8.8)
TNBC – TPS/CPS	NA	​	NA	​	​	​	​	​	​
<1%		0		0	0	0	1 (16.7)	1 (16.7)	2 (5.9)
1%–49%		1 (25.0)		1 (25.0)	0	1 (14.3)	0	1 (16.7)	4 (11.8)
≥50%		0		0	1 (33.3)	0	0	0	1 (2.9)
No PD-L1 test/unknown		0		0	0	0	1 (16.7)	1 (16.7)	2 (5.9)
SCCHN – TPS/CPS	​	​	​	​	NA	​	​	​	​
<1%	0	0	0	0		1 (14.3)	0	0	1 (2.9)
1%–49%	0	1 (25.0)	1 (50.0)	2 (50.0)		1 (14.3)	1 (16.7)	0	6 (17.4)
≥50%	0	0	0	0		1 (14.3)	0	1 (16.7)	2 (5.9)
No PD-L1 test/unknown	1 (50.0)	0	0	0		0	0	0	1 (2.9)
NSCLC mutations^b^	​	​	​	​	​	​	​	​	​
*KRAS*	0	0	0	1 (25.0)	0	1 (14.3)	1 (16.7)	0	3 (8.8)
Other	1 (50.0)	2 (50.0)	0	0	2 (66.7)	3 (42.9)	3 (50.0)	2 (33.3)	13 (38.2)
TNBC mutations^b^	​	​	​	​	​	​	​	​	​
*BRCA2*	0	0	0	0	0	0	1 (16.7)	0	1 (2.9)
Other	0	0	0	1 (25.0)	0	1 (14.3)	2 (33.3)	3 (50.0)	7 (20.6)
SCCHN markers	​	​	​	​	​	​	​	​	​
p16-positive protein expression	0^c^	1 (25.0)	1 (50.0)	2 (50.0)	0	1 (14.3)	1 (16.7)	0	6 (17.6)
Median number of prior anticancer therapies (range)	9.5 (8–11)	5.0 (3–11)	6.5 (3–10)	7.0 (4–11)	9.0 (3–10)	5.0 (3–10)	5.5 (3–10)	6.0 (3–13)	6.0 (3–13)
Prior surgery or procedures	1 (50.0)	3 (75.0)	1 (50.0)	4 (100)	1 (33.3)	4 (57.1)	4 (66.7)	4 (66.7)	22 (64.7)
Prior RT	2 (100)	4 (100)	1 (50.0)	3 (75.0)	2 (66.7)	6 (85.7)	5 (83.3)	5 (83.3)	28 (82.4)

Abbreviations: CPS, combined positive score; TPS, tumor proportion score.

a Patients who received at least one dose of radiation and discontinued the study before receiving dazostinag or pembrolizumab.

b A single patient may have multiple genetic mutations.

c p16 results were not available for one patient who received radiation only.

All 34 patients had received prior anticancer therapy, with a median number of 6 (range, 3–13) prior anticancer therapies; all patients had received prior PD-(L)1 inhibitors. RT was part of prior treatment for 28 (82.4%) patients, and 22 (64.7%) patients had undergone prior surgery (Table 1). At data cutoff, all patients had discontinued the study because of PD (*n* = 24, 70.6%), AEs, symptomatic deterioration (*n* = 4, 11.8% each), withdrawal by patient, or transition to single-patient study to continue receiving dazostinag (*n* = 1, 2.9% each).

### Treatment exposure and DLTs

All patients received RT (three fractions of 8 Gy) without protocol deviations. Radiation could have been administered to multiple tumor locations. The locations for RT were the lung (*n* = 14), lymph node (*n* = 6), liver (*n* = 7), chest wall and bone (*n* = 4 each), skin (*n* = 2), and abdomen, adrenal gland, axilla, back, breast, kidney, right chest wall, spleen, and soft tissues (*n* = 1 each).

Following RT, two patients discontinued the study because of TEAEs, and 32 patients remained and received dazostinag in combination with pembrolizumab across the following dazostinag doses: 0.2 mg (*n* = 4), 0.4 mg (*n* = 2), 0.8 mg (*n* = 4), 1.6 mg (*n* = 3), 2.5 mg (*n* = 7), 3.5 mg (*n* = 6), and 5.0 mg (*n* = 6). No DLTs were reported up to a dose of 5.0 mg of dazostinag, and the maximum tolerated dose (MTD) was not determined. As 5.0 mg was the dose used for cohort expansion in the iintune-1 study (30), there was no further dose escalation. Patients received a median of 6 (range, 2–40) doses of dazostinag (plus pembrolizumab) over a median of 2 (range, 1–14) treatment cycles.

### Safety

The safety population included patients who had received at least one dose of radiation (*n* = 34). Radiation-related TEAEs were reported in 12 patients (35.3%), with fatigue being the most common (*n* = 6, 17.6%); there was one patient with grade 3 radiation-related TEAE (lipase increased) and no radiation-related serious TEAEs. Among the two patients who discontinued the study following RT and before administration of any doses of dazostinag or pembrolizumab, one patient with SCCHN died because of PD, and the second patient with NSCLC had a serious TEAE of grade 3 hypotension. Although emerging after treatment initiation, neither of these events were considered by the investigator to be related to RT. Half of the patients (*n* = 17, 50%) had either dazostinag- or pembrolizumab-related TEAEs, whereas 16 patients (47.1%) reported both dazostinag- and pembrolizumab-related TEAEs.

TEAEs of all causality were observed in all but one patient (*n* = 33; Table 2), with the most common being fatigue (*n* = 18, 52.9%), constipation (*n* = 9, 26.5%), and cough (*n* = 7, 20.6%; Table 3). Fatigue was also the most common dazostinag-related TEAE (*n* = 9, 26.5%), followed by chills (*n* = 3, 8.8%), diarrhea, arthralgia, and myalgia (*n* = 2, 5.9% each; Supplementary Table S4).

**Table 2.** Overview of AEs (safety population).

*n* (%)	Radiation only^a^ *n* = 2	Hypofractionated RT + dazostinag + pembrolizumab							Overall *N* = 34
		Dazostinag 0.2 mg *n* = 4	Dazostinag 0.4 mg *n* = 2	Dazostinag 0.8 mg *n* = 4	Dazostinag 1.6 mg *n* = 3	Dazostinag 2.5 mg *n* = 7	Dazostinag 3.5 mg *n* = 6	Dazostinag 5.0 mg *n* = 6	
Any TEAEs	2 (100)	4 (100)	2 (100)	4 (100)	3 (100)	7 (100)	5 (83.3)	6 (100)	33 (97.1)
Dazostinag-related TEAEs	0	0	2 (100)	2 (50.0)	0	6 (85.7)	4 (66.7)	3 (50.0)	17 (50.0)
Grade ≥3 TEAEs	1 (50.0)	0	1 (50.0)	4 (100)	1 (33.3)	3 (42.9)	2 (33.3)	4 (66.7)	16 (47.1)
Dazostinag-related grade ≥3 TEAEs	0	0	1 (50.0)	1 (25.0)	0	0	0	1 (16.7)	3 (8.8)
Any SAEs	1 (50.0)	0	1 (50.0)	3 (75.0)	1 (33.3)	3 (42.9)	0	3 (50.0)	12 (35.3)
Dazostinag-related SAEs	0	0	0	0	0	0	0	0	0
TEAEs leading to dazostinag dose modification	1 (50.0)	2 (50.0)	1 (50.0)	3 (75.0)	0	4 (57.1)	3 (50.0)	4 (66.7)	18 (52.9)
SAEs leading to dazostinag dose modification	1 (50.0)	0	1 (50.0)	3 (75.0)	0	1 (14.3)	0	1 (16.7)	7 (20.6)
TEAEs leading to dazostinag dose discontinuation	1 (50.0)	1 (25.0)	0	1 (25.0)	0	0	0	1 (16.7)	4 (11.8)
SAEs leading to dazostinag dose discontinuation	1 (50.0)	0	0	1 (25.0)	0	0	0	1 (16.7)	3 (8.8)
Death (on-study)	1 (50.0)	0	0	0	0	0	0	0	1 (2.9)

Abbreviation: SAE, serious AE.

a Patients who received at least one dose of radiation and discontinued the study before receiving dazostinag or pembrolizumab.

**Table 3.** Most common AEs (all-cause) ≥10% overall patients (safety population).

*n* (%)	Radiation only^a^ *n* = 2	Hypofractionated RT + dazostinag + pembrolizumab							Overall *N* = 34
		Dazostinag 0.2 mg *n* = 4	Dazostinag 0.4 mg *n* = 2	Dazostinag 0.8 mg *n* = 4	Dazostinag 1.6 mg *n* = 3	Dazostinag 2.5 mg *n* = 7	Dazostinag 3.5 mg *n* = 6	Dazostinag 5.0 mg *n* = 6	
Fatigue	2 (100)	1 (25.0)	1 (50.0)	3 (75.0)	1 (33.3)	5 (71.4)	3 (50.0)	2 (33.3)	18 (52.9)
Constipation	0	0	1 (50.0)	1 (25.0)	1 (33.3)	4 (57.1)	1 (16.7)	1 (16.7)	9 (26.5)
Cough	0	2 (50.0)	0	0	0	3 (42.9)	1 (16.7)	1 (16.7)	7 (20.6)
Chills	1 (50.0)	1 (25.0)	0	0	0	2 (28.6)	2 (33.3)	0	6 (17.6)
Pyrexia	1 (50.0)	1 (25.0)	1 (50.0)	0	0	2 (28.6)	1 (16.7)	0	6 (17.6)
Diarrhea	1 (50.0)	0	0	0	0	2 (28.6)	3 (50.0)	0	6 (17.6)
Anemia	1 (50.0)	0	1 (50.0)	1 (25.0)	0	0	1 (16.7)	2 (33.3)	6 (17.6)
Headache	0	1 (25.0)	1 (50.0)	0	0	1 (14.3)	1 (16.7)	1 (16.7)	5 (14.7)
Arthralgia	0	1 (25.0)	0	0	0	2 (28.6)	0	2 (33.3)	5 (14.7)
Back pain	0	1 (25.0)	0	1 (25.0)	0	1 (14.3)	1 (16.7)	1 (16.7)	5 (14.7)
Nausea	0	1 (25.0)	1 (50.0)	0	0	2 (28.6)	1 (16.7)	0	5 (14.7)
Vomiting	0	1 (25.0)	0	1 (25.0)	0	1 (14.3)	1 (16.7)	1 (16.7)	5 (14.7)
Pleural effusion	1 (50.0)	0	0	1 (25.0)	0	1 (14.3)	0	1 (16.7)	4 (11.8)
Abdominal pain	1 (50.0)	0	0	1 (25.0)	0	1 (14.3)	1 (16.7)	0	4 (11.8)
Dysphagia	1 (50.0)	0	1 (50.0)	0	0	2 (28.6)	0	0	4 (11.8)
Decreased appetite	0	0	1 (50.0)	1 (25.0)	0	1 (14.3)	1 (16.7)	0	4 (11.8)
Hypoalbuminemia	0	0	1 (50.0)	1 (25.0)	1 (33.3)	0	1 (16.7)	0	4 (11.8)
Pain in extremities	0	0	1 (50.0)	0	0	0	0	3 (50.0)	4 (11.8)
Pruritus	0	1 (25.0)	0	0	0	1 (14.3)	1 (16.7)	1 (16.7)	4 (11.8)

a Patients who received at least one dose of radiation and discontinued the study before receiving dazostinag or pembrolizumab.

Two patients (5.9%) reported immune-related TEAEs: grade 2 optic nerve disorder (TNBC, 3.5 mg dazostinag) and grade 1 to 2 lichenoid keratosis (TNBC, 2.5 mg cohort). Neither of these immune-related TEAEs was considered related to dazostinag, and both events resolved per protocol for TEAEs upon dose reduction and dose delay, respectively. There were no reports of cytokine release syndrome (CRS).

Grade ≥3 TEAEs (all causality) were reported in 16 patients (47.1%), with the most common being anemia in six patients (17.6%). TEAEs associated with grade ≥3 hepatic toxicity were reported in two patients: one patient with SCCHN in the 0.8 mg dazostinag cohort had grade 3 blood bilirubin increased and aspartate aminotransferase (AST) increased, and one patient with NSCLC in the 2.5 mg dazostinag cohort reported grade 3 AST and alanine aminotransferase (ALT) increased. Only the patient with SCCHN had received irradiation to the liver, whereas the patient with NSCLC was irradiated in the lungs and chest wall. Three patients (8.8%) had grade ≥3 TEAEs that were considered related to dazostinag: grade 3 anemia (*n* = 1, 0.4 mg cohort), grade 3 lipase increased (*n* = 1, 0.8 mg cohort), and grade 4 thrombocytopenia (*n* = 1, 5.0 mg cohort). Twelve patients (35.3%) reported serious TEAEs, but none were related to dazostinag or pembrolizumab. Three patients (8.8%) discontinued dazostinag: one because of the aforementioned grade 3 blood bilirubin increased in the 0.8 mg cohort, one because of grade 2 liver function increased (0.2 mg cohort), and one because of grade 3 muscular weakness (5.0 mg cohort). No dazostinag- or pembrolizumab-related deaths were reported (Table 2).

### Clinical responses

Overall, 28 patients were evaluable for response. Four patients discontinued prior to the first posttreatment imaging (AEs *n* = 2, symptomatic deterioration *n* = 1, and patient decision to withdraw *n* = 1). Overall antitumor activity was confirmed in two patients (7.1%): one patient with NSCLC [*KRAS* wild-type grade 3 (poorly differentiated)] who received dazostinag 2.5 mg in combination with pembrolizumab achieved a complete response (CR with a DOR of 9.3 months), and a second patient with SCCHN [p16-negative grade 3 (poorly differentiated) in the nasopharynx] who received dazostinag 5.0 mg in combination with pembrolizumab had a partial response (PR, maintained up to cycle eight/10 months; patient censored before progression or death). Additionally, seven patients (25.0%) had stable disease (SD), the duration of which ranged from 3.0 to 6.5 months: five patients with NSCLC (in the 0.2, 0.4, 0.8, 1.6, and 2.5 mg dazostinag cohorts) and two patients with TNBC (in the 2.5 and 3.5 mg dazostinag cohorts, Table 4; Supplementary Fig. S3A). The clinical benefit rate (CBR; CR + PR + SD for ≥6 months/28 response-evaluable patients) per RECIST v.1.1 criteria was 32.1% (seven SDs for ≥6 months, one CR and one PR).

**Table 4.** Best overall response, per RECIST v.1.1 criteria (response-evaluable population).

*n* (%), unless otherwise stated	Hypofractionated RT + dazostinag + pembrolizumab							Overall *N* = 28
	Dazostinag 0.2 mg *n* = 2	Dazostinag 0.4 mg *n* = 2	Dazostinag 0.8 mg *n* = 4	Dazostinag 1.6 mg *n* = 3	Dazostinag 2.5 mg *n* = 7	Dazostinag 3.5 mg *n* = 6	Dazostinag 5.0 mg *n* = 4	
Best overall response, confirmed	​	​	​	​	​	​	​	​
CR	0	0	0	0	1 (14.3)	0	0	1 (3.6)
PR	0	0	0	0	0	0	1 (25.0)	1 (3.6)
SD	1 (50.0)	1 (50.0)	1 (25.0)	1 (33.3)	2 (28.6)	1 (16.7)	0	7 (25.0)
PD	1 (50.0)	1 (50.0)	3 (75.0)	2 (66.7)	4 (57.1)	5 (83.3)	3 (75.0)	19 (67.9)
ORR, *n* (%), (95% CI)	​	​	​	​	​	​	​	​
Confirmed CR + confirmed PR	0	0	0	0	1 (14.3) (0.4, 57.9)	0	1 (25.0) (0.6, 80.6)	2 (7.1) (0.9, 23.5)

Abbreviations: CI, confidence interval.

Supplementary Tables S5–S7 and Supplementary Fig. S3B and S3C summarize the disease response per the modified itRECIST in overall, irradiated, and nonirradiated lesions. The patient with NSCLC in the 2.5 mg cohort was classified as a CR per both RECIST v.1.1 and itRECIST, whereas the patient with SCCHN in the 5.0 mg cohort was classified as a PR per RECIST v.1.1 and as SD per itRECIST. Additional patients had responses in irradiated lesions only: one patient with NSCLC in the 0.2 mg cohort, classified as SD per RECIST v.1.1, had a PR per itRECIST in irradiated lesions; one patient with TNBC in the 1.6 mg cohort, one patient with SCCHN in the 2.5 mg cohort, and one patient with NSCLC in the 3.5 mg cohort, all classified as PD per RECIST v.1.1, had unconfirmed PRs per itRECIST in irradiated lesions; one patient with TNBC in the 3.5 mg cohort, classified as SD per RECIST v.1.1, also had an unconfirmed PR (later classified as confirmed SD) per itRECIST in irradiated lesions; whereas one patient with NSCLC in the 3.5 mg cohort had confirmed PD per RECIST v.1.1 (following unconfirmed SD) and a confirmed PR per itRECIST in irradiated lesions. According to the modified itRECIST criteria in nonirradiated and irradiated lesions, the CBR was 46.2% and 92.8%, respectively.

### PK

All 32 patients who had received dazostinag plus pembrolizumab were PK-evaluable. The mean (± SD) plasma concentration–time profiles of dazostinag per dosing cohort on C1D1 and C1D15 are shown in Supplementary Fig. S4. Following single intravenous infusion, dazostinag demonstrated dose-dependent PK, with maximal concentrations reached at the EoI.

### Translational and pharmacodynamic markers

**Figure 2.**
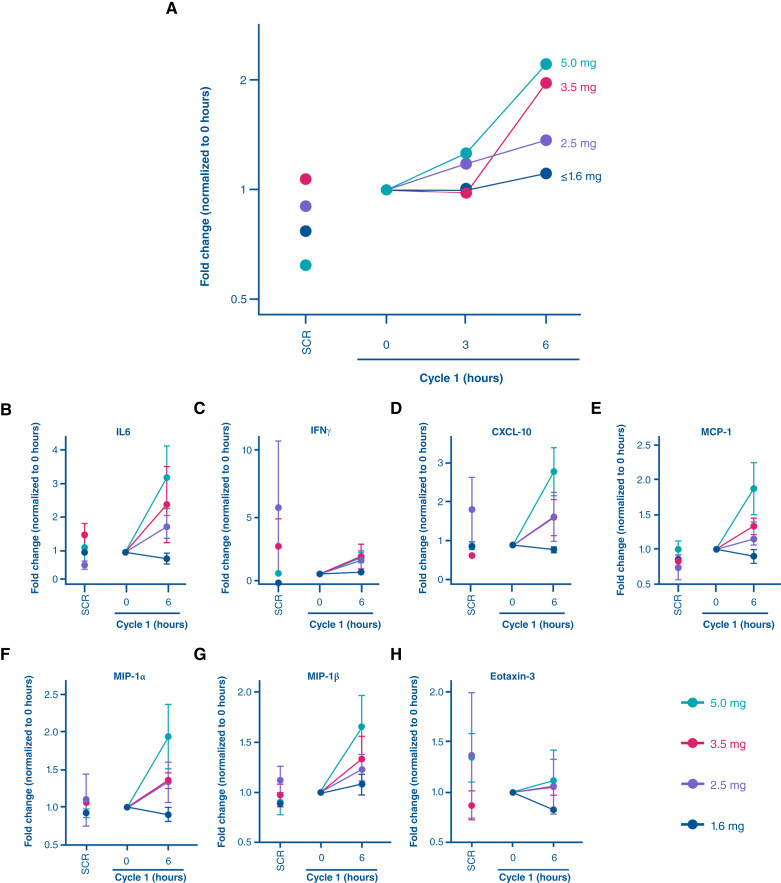
Pharmacodynamic analysis of STING signature and cytokine production in patients treated with dazostinag and pembrolizumab following radiation. **A,** STING signature score fold change by dazostinag doses and normalized to cycle 1, 0 hours. Median fold change across 32 patients per dose per time point plotted. **B–H,** IL6, IFNγ, IP-10, MCP-1, MIP-1α, MIP-1β, and eotaxin-3 cytokines concentration in plasma samples from 21 patients at screening (before radiation) and at 0 and 6 hours after dazostinag administration. Data are shown as the mean fold change (±SEM) normalized to cycle one, 0-hour levels in patients treated with indicated doses of dazostinag and pembrolizumab. CXCL-10, CXC ligand 10.

Blood samples from 32 patients were evaluated for gene expression and cytokine/chemokine changes, based on the previously developed STING gene signature (34). The expression of this gene signature was evaluated in patients at screening, after radiation but prior to dazostinag and pembrolizumab treatment (C1H0), and 6 hours after combination treatment (C1H6). Doses of dazostinag below 1.6 mg did not show meaningful changes at C1H6. At higher doses, the median change (normalized to C1H0) ranged from 1.0- to 2.2-fold for dazostinag doses ranging from 1.6 to 5.0 mg, respectively (Fig. 2A). Cytokine and chemokine changes were also examined at the same time points. Among the panel of analytes examined, IL6, IFNγ, MCP-1, MIP-1α, MIP-1β, and IP-10 demonstrated the most dynamic changes at doses above 1.6 mg (Fig. 2B–H), with maximum induction levels observed with dazostinag 5.0 mg, the highest dose investigated in this study. Overall, the mean change compared with C1H0 increased from ∼1 to 2–3-fold for all analytes, except eotaxin-3 (Fig. 2B–H).

**Figure 3.**
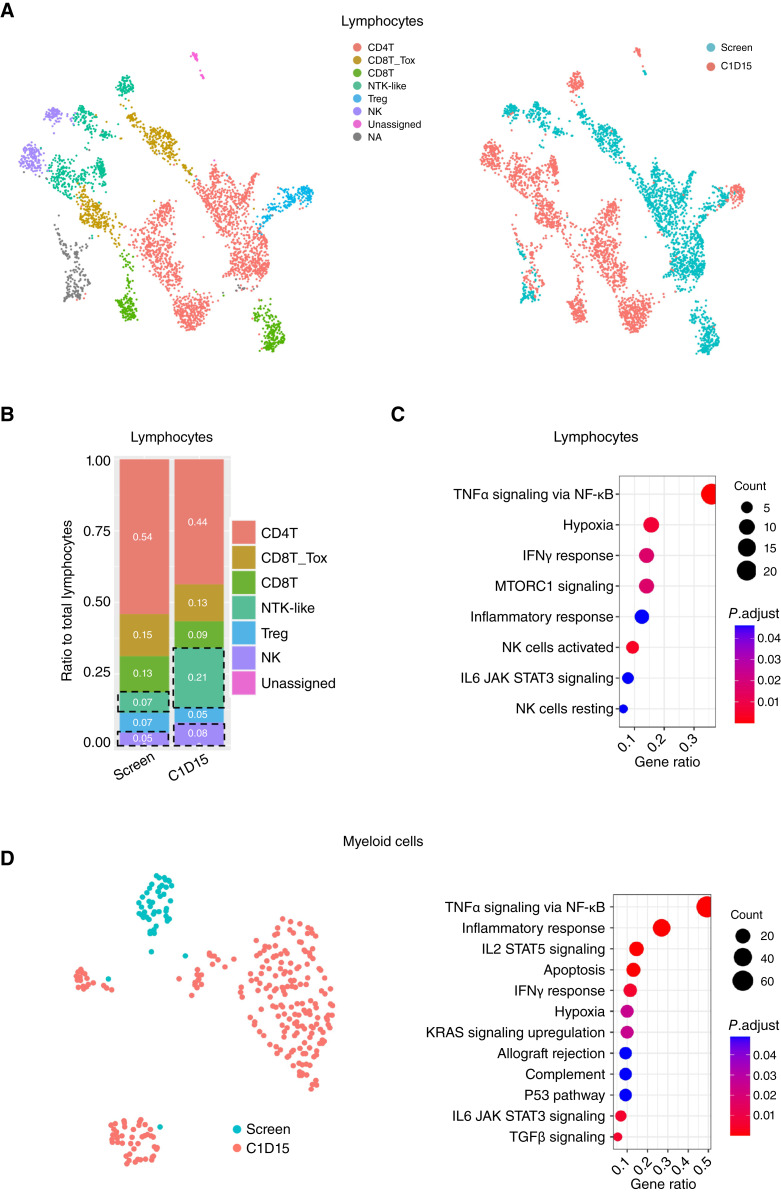
scRNA-seq data from a single patient with tumor biopsy prior to and after dazostinag and pembrolizumab administration following RT. **A,** UMAP of lymphocytes colored by cell type (left) and sampling time (right). **B,** Cell type ratio to total lymphocytes in screen and C1D15 samples, with NK and NKT-like cells highlighted in dashed box. **C,** Hallmark GSEA of upregulated genes in lymphocytes comparing C1D15 with screen samples. **D,** UMAP of myeloid cells colored based on sampling time (left), and Hallmark GSEA of upregulated genes in myeloid cells comparing C1D15 with screen samples (right). CD, cluster of differentiation; KRAS, Kirsten rat sarcoma virus; mTORC1, mTOR complex 1; NA, not applicable; STAT, signal transducer and activator of transcription; Treg, regulatory T cell.

Since checkpoint inhibition enhances immune cell activity, we aimed to understand the impact of STING agonism on CPI treatment by using scRNA-seq data to dissect discrete immune cell subpopulations and signatures in tumors. Due to the complexity of collections and number of resulting evaluable cells, this analysis was limited to a single paired sample set from a patient with NSCLC who received dazostinag (3.5 mg). In this patient, who discontinued treatment after two cycles because of PD, combination therapy increased NK and NKT-like cell abundance by 2.4-fold whereas other populations such as regulatory T cells and CD8 T cells remained unchanged (Fig. 3A and B). Differentially expressed gene analysis was performed comparing lymphocytes from screening and C1D15 samples, and gene set enrichment analysis (GSEA) of significantly upregulated genes was conducted against hallmark gene sets from MSigDB (35, 36). Of the signatures evaluated, TNFα signaling via NF-κB, hypoxia, and IFNγ signatures in lymphocytes showed strong upregulation at C1D15 compared with screening sample (Fig. 3C). Myeloid cell numbers increased following treatment, and hallmark GSEA of myeloid cells showed similar regulated pathways as seen in lymphocytes.

## Discussion

Using an established preclinical tumor-bearing syngeneic mouse model, the combination of dazostinag and fractionated radiation displayed greater tumor regression compared with either treatment alone; however, the addition of an anti–PD-1 antibody did not further enhance the antitumor activity of dazostinag plus RT in this model. This is consistent with previous preclinical studies of single-agent dazostinag (27), and the lack of effect when adding anti–PD-1 may stem from limitations of the preclinical model that was not designed to directly translate to the patient population in CPI-resistant or refractory conditions, rather than a translationally relevant lack of benefit in the clinic. As dazostinag plus radiation was highly efficacious as a doublet combination preclinically, it can be challenging to see additional effect when adding a third agent as the window for enhanced efficacy is reduced. In addition, it is possible that changes to the sequence of treatments or selection of a different tumor model could have provided greater insight into the impact of the triple combination more broadly. Dazostinag and radiation also resulted in induction of peripheral and intratumoral IFNγ and recruitment of proliferating CD8^+^ T cells to tumor, suggestive of an increased adaptive immune response compared with 0-hour C1D1. Overall, these preclinical results provided the rationale for clinical investigation of dazostinag in combination with pembrolizumab following RT in patients with advanced/metastatic NSCLC, TNBC, or SCCHN whose disease had progressed with CPIs.

In the phase 1 clinical study, dazostinag combined with pembrolizumab following hypofractionated RT was well tolerated. When comparing similar doses of dazostinag, the overall safety profile in this study was consistent with the phase 1 iintune-1 study, which assessed dazostinag up to 14.0 mg alone and in combination with pembrolizumab without RT (30). Fatigue was the most frequently reported TEAE (all causality, RT- and dazostinag-related) in this study and in the iintune-1 study (30), consistent with known effects of RT-induced toxicity (37, 38) and immunotherapies (39, 40). The other most common TEAEs associated with dazostinag were flu-like symptoms (chills, diarrhea, arthralgia, and myalgia) which, despite being consistent with an IFN-I–mediated induction of immune response and aligning with the mechanism of action of dazostinag, did not meet the definition of CRS (41). CRS, increasingly assessed as part of immunotherapy trials (42), was not seen in the present study, which is likely due to the low doses of dazostinag (up to 5.0 mg) received. In the iintune-1 study, grade 1 to 2 CRS was reported in 39 patients (27.4%) across all dazostinag doses (ranging from 0.2 to 14.0 mg; ref. 30), with the highest rates observed above 2.5 mg. The only immune-related TEAEs reported in this study were optic nerve disorder and lichenoid keratosis in one patient each; both were of low grade and resolved after dose delay and reduction and were not considered related to dazostinag.

Despite the possibility of adverse reactions in normal tissue due to the known effects of RT (10, 43), we only observed one radiation-related grade 3 TEAE which was lipase increased in a patient in the 5.0 mg dazostinag cohort. RT can affect liver function leading to AEs such as fibrosis (43); in this study, two patients (one with NSCLC and one with SCCHN) reported grade 3 increases in hepatic enzymes (AST, ALT, and blood bilirubin). However, none of these TEAEs were considered by the investigators to be related to RT, and only the patient with SCCHN had received radiation to a lesion in the liver. Taken together, these data suggest that 8 Gy × 3 hypofractionated RT is not associated with any major added toxicities as part of a combination treatment with dazostinag and pembrolizumab.

This study enrolled patients with different solid tumor types who were heavily pretreated, and had previous disease progression on CPIs, with a median number of six prior lines of therapy. Clinical activity according to RECIST v.1.1 criteria was observed in one patient with NSCLC achieving a CR and one patient with SCCHN achieving a PR. Both patients had received immunochemotherapy (including CPI) and respectively reported PR and SD prior to this study. Interestingly, both these patients reported immune-mediated, flu-like TEAEs (such as arthralgia and myalgia), supporting the proposed mechanism of action of RT + CPI. Overall, the eight patients classified as in SD per RECIST v.1.1 had received a median (range) of 3 (1–7) prior lines of therapy, with prior responses including PD, SD, and PR, highlighting a potential clinical benefit in a heavily pretreated patient population.

Overall, the ORR was 7.1% in the present study versus 5.0% in the iintune-1 study (30), with different eligibility criteria (CPI-refractory vs. mix of CPI-naïve and CPI-refractory), study population (limited to NSCLC, TNBC, and SCCHN vs. heterogenous array of solid tumors), dazostinag dosing (up to 5.0 mg vs. up to 14.0 mg), and combination agents (pembrolizumab vs. RT and pembrolizumab; ref. 30). As the dazostinag 5.0 mg dose in combination with pembrolizumab was chosen to be taken forward into expansion cohorts for the iintune-1 study (30), responses at doses <5.0 mg were not expected to be seen this frequently. Given the paucity of published data from clinical trials specifically investigating the combination of RT with pembrolizumab in CPI-exposed populations and acknowledging the limitations of any cross-trial comparisons (particularly of phase 1/2 data with limited patient numbers), evaluation of ORRs between studies should be done with caution. For instance, higher ORRs were reported in other phase 2 trials evaluating RT with pembrolizumab in patients with metastatic NSCLC (36%, PEMBRO-RT, NCT02492568; ref. 18) and metastatic TNBC (17.6%, NCT02730130) regardless of PD-L1 status (15); however, patients in these studies were naïve to CPIs, so a direct comparison cannot be made. Similarly, the ORR was higher in a study investigating pembrolizumab with RT that included patients with a mix of advanced solid tumors (13.2%, NCT02608385; ref. 16); the discrepancy may be attributed to the different numbers or types of prior therapy, patient selection, and disease heterogeneity.

As expected, we observed differences in tumor responses depending on the response assessment; when using the itRECIST criteria for irradiated lesions, six patients with unconfirmed/confirmed PRs by itRECIST were categorized as having PD or SD per RECIST v.1.1. This is consistent with similar trials evaluating pembrolizumab or other CPIs with RT (14–16, 21, 22). One possible explanation is that RT as part of a treatment combination with a CPI and a STING agonist might further increase this clinical response locally. However, further investigation would be needed to fully understand the efficacy of this combination of a STING agonist with immunotherapy following RT and assess the contribution of each treatment.

Patients who received dazostinag (above 2.5 mg) as systemic intravenous injection displayed increased expression of a STING gene signature in peripheral blood and increased levels of IFNγ in plasma, confirming the mechanism of action of dazostinag. Increased production of proinflammatory cytokines such as IL6 and IP-10 was also observed, consistent with an activation of the innate immune system. Pharmacodynamic biomarkers were not shown to correlate with clinical responses in this data set. Findings from the scRNA-seq analysis from one patient’s tumor reported a higher proportion of NKT cells in the immune cell components after treatment; NKT cells have been shown to link innate and adaptive immune systems and play direct and indirect roles in immunosurveillance and antitumor immunity (44). Despite evidence of immune system activation in these patients, clinical activity remained limited, highlighting the complexity and fine balance between immune pathways and tumor regulation.

The investigation of STING agonists, in combination with CPIs, represents an emerging strategy for the treatment of solid tumors. Other studies are currently ongoing to evaluate the safety, tolerability, and recommended dose of STING agonists, administered alone or in combination with CPIs in solid tumors (24, 45–48). Findings from these ongoing trials, along with the data reported here for dazostinag, will help understand how STING agonists can add to combinations involving RT and CPIs.

Limitations of this study include the small number of patients in each dazostinag dosing cohort and within each tumor type across the doses. The dose range of dazostinag also did not exceed 5.0 mg, even though an MTD was not found in this study or the iintune-1 study up to a dose of 14.0 mg; however, pharmacodynamic data confirmed target engagement at the doses investigated, and higher response rates were not seen with higher doses (30). In addition, whereas the exploratory scRNA-seq data provided interesting findings in before versus after treatment changes in immune population subsets, these data were only collected from one patient. This study is also one of the first to report findings from a treatment combination including a STING agonist, a CPI, and RT, therefore making it difficult to contextualize the results.

### Conclusion

Our findings, along with those of the ongoing iintune-1 study, suggest that intravenous dazostinag combined with a CPI with or without hypofractionated RT has an acceptable safety profile and can provide clinical benefit for some patients with advanced/metastatic solid tumors whose disease progressed on CPIs. Further research is needed to understand which patients might benefit the most from this treatment option.

## Supplementary Material

Supplemental Methods Supplementary methods

Supplemental Table S1 Preclinical efficacy study (groups)

Supplemental Table S2 Preclinical pharmacodynamic evaluation (groups)

Supplemental Table S3 Table of representativeness

Supplemental Table S4 Most common dazostinag-related AEs

Supplemental Table S5 Investigator response assessment of best overall response, per modified itRECIST

Supplemental Table S6 Investigator response assessment of irradiated lesions, per modified itRECIST

Supplemental Table S7 Investigator response assessment of non-irradiated lesions, per modified itRECIST

Supplemental Figure S1 Supplementary Figure S1

Supplemental Figure S2 Supplementary Figure S2

Supplemental Figure S3 Supplementary Figure S3

Supplemental Figure S4 Supplementary Figure S4

## Data Availability

The datasets, including the redacted study protocols, redacted statistical analysis plans, and individual participant data supporting the results of the completed study, will be made available upon request to qualified researchers who provide a sound proposal. The data will be provided after its deidentification, in compliance with applicable privacy laws, data protection, and requirements for consent and anonymization. All other data are available in the main manuscript, supplementary files, or upon request from the corresponding author.
