# Supplementary material for: Phase 1b Study of Dazostinag plus Pembrolizumab after Hypofractionated Radiotherapy in Patients with Select Advanced Solid Tumors
Source: Cancer Res Commun. 2025 Dec 31;5(12):2249–63. doi: 10.1158/2767-9764.CRC-25-0566 (PMC12754119; doi:10.1158/2767-9764.CRC-25-0566)
Supplement: Supplemental Methods — Supplementary methods [file crc-25-0566_supplemental_methods_suppsm.pdf]

SUPPLEMENT

Phase 1b Study of Dazostinag Plus Pembrolizumab After Hypofractionated Radiotherapy in Patients With Select Advanced Solid Tumors

Benjamin T. Cooper, Wade T. Iams, David B. Page, Yuan Yuan, Naamit K. Gerber, Jason J. Luke, John P. Gibbs, Richard C. Gregory, Kwok-Kin Wong, Jiehui Deng, Samanthi A. Perera, Kai Ding, Emily R. Roberts, Allison Berger, Camilla L. Christensen, Erica Xin Tong, Angel E. Maldonado López, Vicky A. Appleman, E. Jane Leonard, Alexander Parent, Yu-Chung Huang, Camden Bay, Cong Li, Neil Lineberry, Jeffrey Raizer, Daniel J. Olson, and Steven J. Chmura

SUPPLEMENTAL METHODS

Eligibility criteria

Patient inclusion criteria

Each patient had to meet all the following inclusion criteria to be enrolled in the study:

1. Adult (aged 18 years or older)
2. Voluntary written consent given prior to any study-related procedure not part of standard medical care; consent could have been withdrawn by the patient at any time without any prejudice to future medical care
3. Eastern Cooperative Oncology Group performance status of 0–1
4. Patients must have had at least two measurable Response Evaluation Criteria in Solid Tumors (RECIST) v.1.1-evaluable lesions, with at least one inside and at least one outside of the radiation field. The tumor outside had to be accessible for biopsy at screening and during treatment (which had to be consented by the patient)
5. Patients with pathologically confirmed (cytological diagnosis was adequate) advanced or metastatic non-small cell lung cancer, triple-negative or squamous cell carcinoma of the head and neck who had:
    a. Received or been offered all established standard of care treatment options for which they were eligible, and
    b. Progressed on checkpoint inhibitors (CPIs) in a prior line of therapy
6. Life expectance >12 weeks, as assessed by the investigator
7. Adequate bone marrow, renal and hepatic functions as determined by the following laboratory parameters:
    a. Absolute neutrophil count ≥1000/μL, platelet count ≥75,000/μL, and hemoglobin ≥8.0 g/dL without growth factor support for neutrophils or transfusion support for platelets within 14 days before receiving the first dose of study drug
    b. Total bilirubin ≤1.5x the institutional upper limit of normal (ULN; ≤3.0 mg/dL for patients with Gilbert's disease)
    c. Serum alanine transaminase (ALT) and aspartate transaminase (AST) ≤3x ULN (≤5x ULN for patients with presence of liver metastases)

    d. Albumin ≥3 g/dL
    e. Estimated creatinine clearance ≥30 mL/min (using Cockcroft-Gault formula)
8. Adequate cardiac function, as determined by left-ventricular ejection fraction >50% measured by echocardiogram or multiple-gate acquisition scan within 4 weeks prior to receiving the first dose of study drug
9. Clinically significant toxic effects of previous therapy recovered to grade 1 (per the National Cancer Institute Common Terminology Criteria for Adverse Events v5.0) or baseline; except for alopecia, grade 2 peripheral neuropathy, and/or autoimmune endocrinopathies with stable endocrine replacement therapy
10. Female patients had to be:
    a. Post-menopausal (natural amenorrhea and not due to other medical reason) for at least 1 year prior to screening visit, OR
    b. Surgically sterile, OR
    c. If of child-bearing potential, agreeable to practicing two effective methods of contraception at the same time, from the time of signed informed consent through 120 days after receiving the last dose of the study drug, OR
    d. Agreeable to practice true abstinence, when this was in line with the preferred and usual lifestyle of the patient
        i. Note: periodic abstinence (e.g. calendar, ovulation, symptothermal, post-ovulation methods), withdrawal, spermicides only, and lactation amenorrhea were not acceptable methods of contraception
11. Male patients, even surgically sterilized, had to:
    a. Agree to practice effective barrier contraception during the entire study treatment period and through 120 after receiving the last dose of the study drug, OR
    b. Agree to practice true abstinence, when this was in line with the preferred and usual lifestyle of the patient

Patient exclusion criteria

1. History of any of the following ≤6 months prior to receiving the first dose of the study drug: congestive heart failure (as per the New York Heart Association Grade III or IV), unstable angina, myocardial infarction, persistent hypertension (≥160/100 mm-Hg despite optimal medical therapy), ongoing cardiac arrythmias of grade >2 (including atrial flutter/fibrillation or intermittent ventricular tachycardia), other ongoing serious cardiac conditions (e.g. grade 3 pericardial effusion or grade 3 restrictive cardiomyopathy) or symptomatic cerebrovascular events. Chronic, stable atrial fibrillation on stable anticoagulation therapy, including low-molecular-weight heparin, was allowed
2. History of brain metastasis, unless clinically stable (i.e. treatment completed ≥4 weeks prior) following prior surgery, whole-brain radiation or stereotactic radiosurgery, AND off corticosteroids
3. Known history of uncontrolled autoimmune disorders, HIV infection or other relevant congenital or acquired immunodeficiencies
4. Chronic, active hepatitis (e.g. patients with known hepatitis B surface antigen seropositive and/or detectable hepatitis C virus). Patients with positive hepatitis B core antibody could be enrolled but must have undetectable serum hepatitis B virus DNA. Patients with positive hepatitis C virus antibody have to have undetectable hepatitis C virus RNA serum levels
5. Contraindication and/or history of intolerance to CPI administration
6. Contraindication and/or history of intolerance to radiotherapy
7. Any illness, metabolic dysfunction, physical examination, or clinical laboratory findings that gave reasonable suspicion of a disease or condition that would contraindicate the use of an investigational drug or that would limit compliance with study requirements or compromise ability to provide written informed consent
8. Treatment with any investigational products and systemic anticancer drugs, including vascular endothelial growth factor inhibitors, within 14 days or five half-lives, whichever was shorter, before cycle 1 day 1 (C1D1) of receiving study drugs
9. Concurrent chemotherapy, immunotherapy (except for pembrolizumab), biologic or hormonal therapy (except for adjuvant endocrine therapy for

history of breast cancer). Concurrent use of hormones for non-cancer-related conditions was acceptable

10. Prior radiation to lesions chosen for biopsy or response assessment
11. Prior radiation to lesions other than those chosen for radiotherapy or biopsy within 4 weeks of C1D1
12. Use of systemic corticosteroids, or other immunosuppressive therapy, concurrently or within 14 days of start of radiotherapy, with the exceptions:
    a. Topical, intranasal, inhaled, ocular and/or intra-articular corticosteroids
    b. Physiological doses of replacement steroid therapy (e.g. for adrenal insufficiency)
13. Receipt of live attenuated vaccine within 28 days of C1D1
14. Recipient of allogeneic or autologous stem cell transplant or organ transplantation
15. Female patients who were lactating (and chose not to discontinue breastfeeding), or had positive serum pregnancy test during screening period or positive urine pregnancy test on day 1 before receiving the first dose of the study drug
16. Ongoing grade ≥2 infection or patients with grade ≥2 fever of malignant origin
17. QT intervals corrected for heart rate using Frederica’s (cube root) correction >450 msec for males or >475 msec for females on a 12-lead electrocardiogram during screening period
18. Grade ≥2 hypotension at screening or during C1D1 pre-dose assessment
19. Oxygen saturation <92% on room air at screening or during C1D1 pre-dose assessment
20. Use of medication that are known clinical organic anion transporting polypeptide (OATP)1B1 or OATP1B3 inhibitors, concurrently or within 14 days of C1D1
21. Patients treated with other stimulator of interferon genes agonist/antagonist and toll-like receptors agonists within the past 6 months
22. Current smoker or vaping within 90 days of C1D1
23. Current diagnosis of pneumonitis, interstitial lung disease, severe chronic obstructive pulmonary disease, idiopathic pulmonary fibrosis, other restrictive lung disease, acute pulmonary embolism, or grade ≥2 pleural effusion or ascites not controlled by tap or requiring indwelling catheters

Definition of events and populations

DLT

A dose-limiting toxicity (DLT) was defined as any of the following treatment-emergent adverse events (TEAEs) that occurred during the first cycle and were considered by the investigator to be at least possibly related to dazostinag in combination with pembrolizumab:

- Any grade 5 TEAE
- Grade 4 anemia
- Grade ≥4 neutropenia lasting ≥7 days, or requiring the use of granulocyte colony stimulating factor
- Any febrile neutropenia
- Platelet count <10,000/μL at any time
- Grade 4 thrombocytopenia lasting ≥7 days, or grade ≥3 thrombocytopenia associated with clinically significant bleeding
- Grade ≥3 cytokine release syndrome
- Grade ≥2 immune-mediated uveitis that did not respond to topical therapy and did not improve to grade ≤1 severity within 2 weeks of initiation of topical therapy OR required systemic treatment
- Delay in initiation of cycle 2 by more than 21 days from the calculated start date due to lack of recovery of treatment-related hematological or non-hematological toxicities
- Development of grade ≥3 myelitis, pneumonitis, gastritis, hepatitis, dermatitis or pain flare in the relevant radiation field
- Ang grade ≥3 non-hematologic toxicity with the following exceptions:
    - Grade 3 arthralgia/myalgia that responds to non-steroidal anti-inflammatory medication within 1 week
    - Grade 3 fatigue lasting <7 days
    - Any grade 3 endocrinopathy that was adequately controlled by hormonal replacement
    - Grade 3 or 4 inflammatory reaction attributed to a local antitumor response (defined as local pain, irritation or rash localized at sites of known/suspected tumor)

- Transient (≤24 hours) grade 3 flu-like symptoms that resolved spontaneously or were controlled with medical management
- Grade 3 or 4 asymptomatic laboratory changes (other than renal and hepatic laboratory values) that were successfully corrected (to grade ≤1 or baseline) within 72 hours
- Isolated elevation of ALT and/or AST (≤10x ULN) in the absence of significant bilirubin elevation (grade <3), excluding elevation meeting Hy's Law
- Grade 3 nausea and/or emesis that were controlled to grade ≤1 within 3 days with the use of antiemetics (such as metoclopramide, prochlorperazine, 5-hydroxytryptamine [serotonin] type 3 receptor antagonist and/or neurokinin-1 receptor antagonist)
- Grade 3 rash and pruritis that responded to standard treatment and resolved or improved to grade <3 within 7 days
- Grade 3 diarrhea that was controlled to grade ≤2 within 3 days with supportive treatment
- Alopecia

Populations

Safety population: patients who had received at least one dose of radiation.

Pharmacokinetic analysis population: patients who received at least one dose of dazostinag and had plasma concentration data collected.

DLT-evaluable population: patients who received all cycle 1 doses of dazostinag with pembrolizumab and radiation without experiencing a DLT event, or who had a DLT during cycle 1. Patients who received only one or two doses of radiation were not considered DLT-evaluable and could be replaced within the same cohort but were allowed to stay on study.

Response-evaluable population: subset of the safety population including patients with measurable disease at baseline and at least one post-treatment evaluation.

Pharmacodynamic population: patients in the safety population who received at least one dose of dazostinag and had baseline and at least one post-baseline pharmacodynamic sample assessment.
