# Supplementary material for: Phase 1b Study of Dazostinag plus Pembrolizumab after Hypofractionated Radiotherapy in Patients with Select Advanced Solid Tumors
Source: Cancer Res Commun. 2025 Dec 31;5(12):2249–63. doi: 10.1158/2767-9764.CRC-25-0566 (PMC12754119; doi:10.1158/2767-9764.CRC-25-0566)
Supplement: Supplemental Table S1 — Preclinical efficacy study (groups) [file crc-25-0566_supplemental_table_s1_suppst1.pdf]

**Supplemental Table S1** Preclinical efficacy study (groups)

| Group | *N* | Treatment | Dose | ROA | Regimen | Days of treatment |
|---|---|---|---|---|---|---|
| **1** | 8 | Vehicle | 0.2 mL/20 g | IV | Q3Dx3 | 0, 3 |
| | | Isotype control (Clone 2A3) | 10.0 mg/kg | IP | Q3Dx3 | 0, 3 |
| | | Mock radiation | NA | NA | QDx3 | -4 – -2 |
| **2** | 8 | Dazostinag | 1.0 mg/kg | IV | Q3Dx3 | 0, 3 |
| | | Isotype control (Clone 2A3) | 10.0 mg/kg | IP | Q3Dx3 | 0, 3 |
| | | Mock radiation | NA | NA | QDx3 | -4 – -2 |
| **3** | 8 | Dazostinag | 0.25 mg/kg | IV | Q3Dx3 | 0, 3 |
| | | Isotype control (Clone 2A3) | 10.0 mg/kg | IP | Q3Dx3 | 0, 3 |
| | | Mock radiation | NA | NA | QDx3 | -4 – -2 |
| **4** | 8 | Vehicle | 0.2 mL/20 g | IV | Q3Dx3 | 0, 3 |
| | | Anti-mPD-1 (Clone RMP1-14) | 10.0 mg/kg | IP | Q3Dx3 | 0, 3 |
| | | Mock radiation | NA | NA | QDx3 | -4 – -2 |
| **5** | 8 | Vehicle | 0.2 mL/20 g | IV | Q3Dx3 | 0, 3 |
| | | Isotype control (Clone 2A3) | 10.0 mg/kg | IP | Q3Dx3 | 0, 3 |
| | | Radiation | 8 Gy | SARRP | QDx3 | -4 – -2 |
| **6** | 8 | Dazostinag | 1 mg/kg | IV | Q3Dx3 | 0, 3 |
| | | Isotype control (Clone 2A3) | 10.0 mg/kg | IP | Q3Dx3 | 0, 3 |
| | | Radiation | 8 Gy | SARRP | QDx3 | -4 – -2 |
| **7** | 8 | Vehicle | 0.2 mL/20 g | IV | Q3Dx3 | 0, 3 |
| | | Anti-mPD-1 (Clone RMP1-14) | 10.0 mg/kg | IP | Q3Dx3 | 0, 3 |
| | | Radiation | 8 Gy | SARRP | QDx3 | -4 – -2 |
| **8** | 8 | Dazostinag | 1.0 mg/kg | IV | Q3Dx3 | 0, 3 |
| | | Anti-mPD-1 (Clone RMP1-14) | 10.0 mg/kg | IP | Q3Dx3 | 0, 3 |
| | | Radiation | 8 Gy | SARRP | QDx3 | -4 – -2 |
| **9** | 8 | Dazostinag | 0.25 mg/kg | IV | Q3Dx3 | 0, 3 |
| | | Anti-mPD-1 (Clone RMP1-14) | 10.0 mg/kg | IP | Q3Dx3 | 0, 3 |
| | | Radiation | 8 Gy | SARRP | QDx3 | -4– -2 |

Gy, Gray; IP, intraperitoneal; IV, intravenous; mPD-1, monoclonal programed death protein 1; NA, not applicable; Q3Dx3, once every 3 days for 3 instances; QDx3, daily for 3 instances; ROA, route of administration; SARRP, Small Animal Radiation Research Platform.
