# Supplementary material for: Phase 1b Study of Dazostinag plus Pembrolizumab after Hypofractionated Radiotherapy in Patients with Select Advanced Solid Tumors
Source: Cancer Res Commun. 2025 Dec 31;5(12):2249–63. doi: 10.1158/2767-9764.CRC-25-0566 (PMC12754119; doi:10.1158/2767-9764.CRC-25-0566)
Supplement: Supplemental Table S2 — Preclinical pharmacodynamic evaluation (groups) [file crc-25-0566_supplemental_table_s2_suppst2.pdf]

**Supplemental Table S2** Preclinical pharmacodynamic evaluation (groups)

| Group | N | Treatment | Dose | ROA | Regimen | Drug treatment day(s) | Sample collection day(s) |
|---|---|---|---|---|---|---|---|
| **1** | 6 | Mock radiation | 0 Gy | NA | QDx3 | -4 – -2 | -1 |
| **2** | 6 | Mock radiation | 0 Gy | NA | QDx3 | -4 – -2 | -1 |
| | | PBS | 0.2 mL/20g | IV | QDx1 | 0 | 1, 5 |
| **3** | 6 | Mock radiation | 0 Gy | NA | QDx3 | -4 – -2 | -1 |
| | | PBS | 0.2 mL/20g | IV | Q3Dx2 | 0, 3 | 1, 5 |
| **4** | 6 | Radiation | 8 Gy | SARRP | QDx3 | -4 – -2 | -1 |
| **5** | 6 | Radiation | 8 Gy | SARRP | QDx3 | -4 – -2 | -1 |
| | | PBS | 0.2 mL/20g | IV | QDx1 | 0 | 1, 5 |
| **6** | 6 | Radiation | 8 Gy | SARRP | QDx3 | -4 – -2 | -1 |
| | | PBS | 0.2 mL/20g | IV | Q3Dx2 | 0, 3 | 1, 5 |
| **7** | 12 | Mock radiation | 0 Gy | NA | QDx3 | -4 – -2 | -1 |
| | | Dazostinag | 1.0 mg/kg | IV | QDx1 | 0 | 1, 5 |
| **8** | 6 | Mock radiation | 0 Gy | NA | QDx3 | -4 – -2 | -1 |
| | | Dazostinag | 1.0 mg/kg | IV | Q3Dx2 | 0, 3 | 1, 5 |
| **9** | 6 | Radiation | 8 Gy | SARRP | QDx3 | -4 – -2 | -1 |
| | | Dazostinag | 1.0 mg/kg | IV | QDx1 | 0 | 1, 5 |
| **10** | 6 | Radiation | 8 Gy | SARRP | QDx3 | -4 – -2 | -1 |
| | | Dazostinag | 1.0 mg/kg | IV | Q3Dx2 | 0, 3 | 1, 5 |

Gy, Gray; IV, intravenous; NA, not applicable; PBS, phosphate-buffered saline; Q3Dx1/2, once every 3 days for 1/2 instances; QDx3, daily for 3 instances; ROA, route of administration; SARRP, Small Animal Radiation Research Platform.
