# Supplementary material for: Phase 1b Study of Dazostinag plus Pembrolizumab after Hypofractionated Radiotherapy in Patients with Select Advanced Solid Tumors
Source: Cancer Res Commun. 2025 Dec 31;5(12):2249–63. doi: 10.1158/2767-9764.CRC-25-0566 (PMC12754119; doi:10.1158/2767-9764.CRC-25-0566)
Supplement: Supplemental Table S3 — Table of representativeness [file crc-25-0566_supplemental_table_s3_suppst3.pdf]

Supplemental Table S3 Table of representativeness

Cancer type	Non-small cell lung cancer (NSCLC)	Triple-negative breast cancer (TNBC)	Squamous cell carcinoma of the head and neck (SCCHN)
**Considerations relating to:**			
**Sex**	• In the US, the lifetime risk of developing lung cancer is approximately 1 in 17 for men and 1 in 18 for women[1]<br>- NSCLC accounts for around 87% of all lung cancer cases[1]<br>Although incidence rates remain lower in women, the disparity between sexes is narrowing[1]	• Fewer than 1% of breast carcinomas occur in men[5]<br>Approximately 95% of male breast cancers are hormone receptor positive; TNBC is particularly rare in men[5]	In the US, SCCHN is approximately three times more common in men than women[12,13]
**Age**	Most individuals diagnosed with lung cancer are >65 years; the average age of diagnosis in the US is around 70 years[1]	TNBC tends to be more common in women who are younger than 40 years old[6]	The median age at diagnosis in the US is 64 years; around half of patients are diagnosed between the ages of 55 and 74 years[13]
**Race/Ethnicity**	• In the US, Black men are approximately 12% more likely to develop lung cancer than White men[1]<br>The rate of lung cancer is approximately 16% lower in Black women than in White women[1]	Black women have been reported to be 2.7 times more likely to be diagnosed with TNBC than White women[7]	• In the US, among men, the incidence of SCCHN is highest in non-Hispanic White (20.1/100,000) and American Indian and Alaska Native (17.5/100,000) patients, and lowest in Hispanic (10.3/100,000) and Asian/Pacific Islander (12.0/100,000) patients[13]

			Among females, the incidence is highest in non-Hispanic White (7.1/100,000) and Asian/Pacific Islander (5.0/100,000) patients, and lowest in Hispanic (4.3/100,000) and Black (5.0/100,000) patients[13]
**Geography**	There are US state-level disparities in lung cancer incidence with lower rates seen in the West (41.7/100,000 in 2020) versus the Northeast (60.7/100,000), Midwest (67.1/100,000), and the South (61.3/100,000)[2]	Substantial state-level variations in racial and ethnic disparities in TNBC incidence rates have been reported, with Black women in Delaware, Missouri, Louisiana, and Mississippi having the highest rates among all states and racial and ethnic populations[8]	SCCHN incidence rate varies across US states with the highest rates reported in West Virginia (14.4/100,000) and the lowest rates in Utah (9.4/100,000)[14]
**Other considerations**	• Tobacco smoke is the predominant risk factor for developing lung cancer; in the US cigarette smoke is linked to approximately 80–90% of lung cancer deaths[3]<br>Other risk factors in the US include exposure to radon, asbestos, diesel exhaust, or ionizing radiation; a personal or family history of lung cancer; or a diagnosis of chronic obstructive pulmonary disease[3,4]	• BRCA1 mutation:<br>- In the US, an estimated 13% of women in the general population will develop breast cancer; for woman with a BRCA1 mutation, it is estimated that >60% will develop breast cancer[9]<br>Most BRCA1-associated breast cancers (>75%) are triple-negative[10,11]	• Tobacco smoke is a leading risk factor, accounting for approximately 75% of SCCHN cases in Western Europe[13]<br>Human Papillomavirus accounts for 72% of SCCHN cases in developed nations compared with 13% in developing nations[13]

**Overall representativeness of this study**	• The current study included patients with NSCLC (n=15; 44%), TNBC (n=9; 26%), and SCCHN (n=10; 29%). Baseline demographics are reported for the overall study population<br>○ The median patient age in the current study was 61 years<br>○ Just over half of the population (56%) were male<br>○ In total, 71% of the patients in the current study were White and 15% were Black/African American<br><br>This is a phase 1 study with a small number of patients (n=34) across three different indications, resulting in the study population not aligned with the typical cancer patient demographics.

References:

1. American Cancer Society (2025). Key statistics for lung cancer. Accessed July 11, 2025. Available from: https://www.cancer.org/cancer/types/lung-cancer/about/key-statistics.html.
2. Bryant-Genevier J, Kava CM, Melkonian SC, Siegel DA. State and regional trends in incidence and early detection of lung cancer among US adults, 2010–2020. Prev Chronic Dis 2024;21:240016.
3. US Centers for Disease Control and Prevention (2025). Lung cancer risk factors. Accessed July 11, 2025. Available from: https://www.cdc.gov/lung-cancer/risk-factors/index.html.
4. World Health Organization (2024). Chronic obstructive pulmonary disease (COPD). Accessed July 11, 2025. Available from: https://www.who.int/news-room/fact-sheets/detail/chronic-obstructive-pulmonary-disease-%28copd%29.
5. Qavi Q, Alkistawi F, Kumar S, Ahmed R, Saad Abdalla Al-Zawi A. Male triple-negative breast cancer. Cureus 2021;13(4):e14542.
6. American Cancer Society (2025). Triple-negative breast cancer. Accessed July 11, 2025. Available from: https://www.cancer.org/cancer/types/breast-cancer/about/types-of-breast-cancer/triple-negative.html.
7. McCarthy AM, Friebel-Klingner T, Ehsan S, He W, Welch M, Chen J, et al. Relationship of established risk factors with breast cancer subtypes. Cancer Med 2021;10(18):6456–67.
8. Sung H, Wiese D, Jatoi I, Jemal A. State variation in racial and ethnic disparities in incidence of triple-negative breast cancer among US women. JAMA Oncol 2023;9(5):700–4.
9. National Cancer Institute (2024). BRCA gene changes: cancer risk and genetic testing. Accessed July 11, 2025. Available from: https://www.cancer.gov/about-cancer/causes-prevention/genetics/brca-fact-sheet#:~:text=Female%20breast%20cancer%3A%20More%20than%2060%25%20of%20women,population%20will%20develop%20breast%20cancer%20during%20their%20lifetime.

10. Anders C, Carey LA. Understanding and treating triple-negative breast cancer. Oncology (Williston Park) 2008;22(11):1233–43.
11. Ballatore Z, Pistelli M, Bracci R, Bianchi F, Maccaroni E, Belvederesi L, et al. Triple-negative breast cancer and BRCA mutation: looking at the future. Ann Oncol 2016;27(Suppl_6_):vi50.
12. US Centers for Disease Control and Prevention (2025). United States cancer statistics: data visualization. Oral cavity and pharynx. Accessed July 11, 2025. Available from: https://gis.cdc.gov/Cancer/USCS/#/special-topic/demographics?datatype=1&cancer=18&indicator=value&timeperiod=1&sexes=1_2_3&races=1&ages=23&tab=2&view=chart&xaxis=sexes.
13. Barsouk A, Aluru JS, Rawla P, Saginala K, Barsouk A. Epidemiology, risk factors, and prevention of head and neck squamous cell carcinoma. Med Sci (Basel) 2023;11(2):42.
14. National Cancer Institute. State cancer rates 2017–2021. Oral cavity & pharynx (all stages). Accessed July 11, 2025. Available from: https://statecancerprofiles.cancer.gov/incidencerates/index.php?stateFIPS=00&areatype=state&cancer=003&race=00&sex=0&age=001&stage=999&year=0&type=incd&sortVariableName=rate&sortOrder=default&output=0#results.
