# Supplementary material for: Phase 1b Study of Dazostinag plus Pembrolizumab after Hypofractionated Radiotherapy in Patients with Select Advanced Solid Tumors
Source: Cancer Res Commun. 2025 Dec 31;5(12):2249–63. doi: 10.1158/2767-9764.CRC-25-0566 (PMC12754119; doi:10.1158/2767-9764.CRC-25-0566)
Supplement: Supplemental Table S4 — Most common dazostinag-related AEs [file crc-25-0566_supplemental_table_s4_suppst4.pdf]

**Supplemental Table S4** Most common dazostinag-related AEs

| *n* (%) | Radiation only[a] *n* = 2 | Hypofractionated radiotherapy + dazostinag + pembrolizumab | | | | | | | Overall *N* = 34 |
|---|---|---|---|---|---|---|---|---|---|
| | | Dazostinag 0.2 mg *n* = 4 | Dazostinag 0.4 mg *n* = 2 | Dazostinag 0.8 mg *n* = 4 | Dazostinag 1.6 mg *n* = 3 | Dazostinag 2.5 mg *n* = 7 | Dazostinag 3.5 mg *n* = 6 | Dazostinag 5.0 mg *n* = 6 | |
| **Fatigue** | 0 | 0 | 1 (50.0) | 1 (25.0) | 0 | 3 (42.9) | 3 (50.0) | 1 (16.7) | 9 (26.5) |
| **Chills** | 0 | 0 | 0 | 0 | 0 | 1 (14.3) | 2 (33.3) | 0 | 3 (8.8) |
| **Diarrhea** | 0 | 0 | 0 | 0 | 0 | 1 (14.3) | 1 (16.7) | 0 | 2 (5.9) |
| **Arthralgia** | 0 | 0 | 0 | 0 | 0 | 1 (14.3) | 0 | 1 (16.7) | 2 (5.9) |
| **Myalgia** | 0 | 0 | 0 | 0 | 0 | 1 (14.3) | 0 | 1 (16.7) | 2 (5.9) |
| **Pyrexia** | 0 | 0 | 1 (50.0) | 0 | 0 | 0 | 0 | 0 | 1 (2.9) |
| **Stomatitis** | 0 | 0 | 0 | 0 | 0 | 0 | 1 (16.7) | 0 | 1 (2.9) |
| **Vomiting** | 0 | 0 | 0 | 1 (25.0) | 0 | 0 | 0 | 0 | 1 (2.9) |
| **Anemia** | 0 | 0 | 1 (50.0) | 0 | 0 | 0 | 0 | 0 | 1 (2.9) |
| **Thrombocytopenia** | 0 | 0 | 0 | 0 | 0 | 0 | 0 | 1 (16.7) | 1 (2.9) |
| **Hyperhidrosis** | 0 | 0 | 0 | 0 | 0 | 0 | 1 (16.7) | 0 | 1 (2.9) |
| **Lichenoid keratosis** | 0 | 0 | 0 | 0 | 0 | 1 (14.3) | 0 | 0 | 1 (2.9) |
| **Pruritus** | 0 | 0 | 0 | 0 | 0 | 0 | 0 | 1 (16.7) | 1 (2.9) |
| **Rash** | 0 | 0 | 0 | 0 | 0 | 1 (14.3) | 0 | 0 | 1 (2.9) |
| **Rash erythematous** | 0 | 0 | 0 | 0 | 0 | 1 (14.3) | 0 | 0 | 1 (2.9) |
| **Pain in extremities** | 0 | 0 | 0 | 0 | 0 | 0 | 0 | 1 (16.7) | 1 (2.9) |

| | | | | | | | | | |
|---|---|---|---|---|---|---|---|---|---|
| **Dizziness** | 0 | 0 | 0 | 0 | 0 | 1 (14.3) | 0 | 0 | 1 (2.9) |
| **Dysgeusia** | 0 | 0 | 0 | 0 | 0 | 0 | 0 | 1 (16.7) | 1 (2.9) |
| **Hypothyroidism** | 0 | 0 | 0 | 0 | 0 | 1 (14.3) | 0 | 0 | 1 (2.9) |
| **Optic nerve disorder** | 0 | 0 | 0 | 0 | 0 | 0 | 1 (16.7) | 0 | 1 (2.9) |
| **Infusion-related reaction** | 0 | 0 | 0 | 1 (25.0) | 0 | 0 | 0 | 0 | 1 (2.9) |
| **Amylase increased** | 0 | 0 | 0 | 1 (25.0) | 0 | 0 | 0 | 0 | 1 (2.9) |
| **Lipase increased** | 0 | 0 | 0 | 0 | 0 | 0 | 0 | 0 | 1 (2.9) |
| **Decreased appetite** | 0 | 0 | 0 | 0 | 0 | 0 | 1 (16.7) | 0 | 1 (2.9) |
| **Cough** | 0 | 0 | 0 | 0 | 0 | 1 (14.3) | 0 | 0 | 1 (2.9) |
| **Pneumonitis** | 0 | 0 | 0 | 0 | 0 | 1 (14.3) | 0 | 0 | 1 (2.9) |
| **Hypotension** | 0 | 0 | 0 | 0 | 0 | 0 | 1 (16.7) | 0 | 1 (2.9) |

Abbreviations: AE, adverse event.
[a]Patients who received at least one dose of radiation and discontinued the study before receiving dazostinag or pembrolizumab.
