# Supplementary material for: Phase 1b Study of Dazostinag plus Pembrolizumab after Hypofractionated Radiotherapy in Patients with Select Advanced Solid Tumors
Source: Cancer Res Commun. 2025 Dec 31;5(12):2249–63. doi: 10.1158/2767-9764.CRC-25-0566 (PMC12754119; doi:10.1158/2767-9764.CRC-25-0566)
Supplement: Supplemental Table S6 — Investigator response assessment of irradiated lesions, per modified itRECIST [file crc-25-0566_supplemental_table_s6_suppst6.pdf]

Supplemental Table S6 Investigator response assessment of irradiated lesions, per modified itRECIST

	Hypofractionated radiotherapy + dazostinag + pembrolizumab							
***n* (%), unless otherwise stated**	**Dazostinag 0.2 mg *n* = 2**	**Dazostinag 0.4 mg *n* = 2**	**Dazostinag 0.8 mg *n* = 4**	**Dazostinag 1.6 mg *n* = 3**	**Dazostinag 2.5 mg *n* = 7**	**Dazostinag 3.5 mg *n* = 6**	**Dazostinag 5.0 mg *n* = 4**	**Overall N = 28**
**Best overall response, confirmed**								
Complete response	0	0	0	0	1 (14.3)	0	0	1 (3.6)
Partial response	1 (50.0)	0	0	0	0	1 (16.7)	0	2 (7.1)
Stable disease	0	2 (100)	2 (50.0)	1 (33.3)	2 (28.6)	2 (33.3)	1 (25.0)	10 (35.7)
Progressive disease	0	0	1 (25.0)	0	0	0	0	1 (3.6)
Not evaluable	1 (50.0)	0	1 (25.0)	2 (66.7)	4 (57.1)	3 (50.0)	3 (75.0)	14 (50.0)
**ORR, n (%), (95% CI)**								
Confirmed complete response + confirmed partial response	1 (50.0) (1.3, 98.7)	0	0	0	1 (14.3) (0.4, 57.9)	1 (16.7) (0.4, 64.1)	0	3 (10.7) (2.3, 28.2)

CI, confidence interval; ORR, overall response rate; itRECIST, Response Criteria for Intratumoral Immunotherapy in Solid Tumors.
