# Supplementary material for: Phase 1b Study of Dazostinag plus Pembrolizumab after Hypofractionated Radiotherapy in Patients with Select Advanced Solid Tumors
Source: Cancer Res Commun. 2025 Dec 31;5(12):2249–63. doi: 10.1158/2767-9764.CRC-25-0566 (PMC12754119; doi:10.1158/2767-9764.CRC-25-0566)
Supplement: Supplemental Table S7 — Investigator response assessment of non-irradiated lesions, per modified itRECIST [file crc-25-0566_supplemental_table_s7_suppst7.pdf]

Supplemental Table S7 Investigator response assessment of non-irradiated lesions, per modified itRECIST

	Hypofractionated radiotherapy + dazostinag + pembrolizumab							
***n* (%), unless otherwise stated**	**Dazostinag 0.2 mg *n* = 2**	**Dazostinag 0.4 mg *n* = 2**	**Dazostinag 0.8 mg *n* = 4**	**Dazostinag 1.6 mg *n* = 3**	**Dazostinag 2.5 mg *n* = 7**	**Dazostinag 3.5 mg *n* = 6**	**Dazostinag 5.0 mg *n* = 4**	**Overall *N* = 28**
**Best overall response, confirmed**								
Complete response	0	0	0	0	1 (14.3)	0	0	1 (3.6)
Partial response	0	0	0	0	0	0	1 (25.0)	1 (3.6)
Stable disease	1 (50.0)	2 (100)	1 (25.0)	1 (33.3)	2 (28.6)	2 (33.3)	0	9 (32.6)
Progressive disease	1 (50.0)	0	3 (75.0)	2 (66.7)	3 (42.9)	4 (66.7)	2 (50.0)	15 (53.6)
Not evaluable	0	0	0	0	1 (14.3)	0	1 (25.0)	2 (7.1)
**ORR, n (%), (95% CI)**								
Confirmed complete response + confirmed partial response	0	0	0	0	1 (14.3) (0.4, 57.9)	0	1 (25.0) (0.6, 80.6)	2 (7.1) (0.9, 23.5)

CI, confidence interval; ORR, overall response rate; itRECIST, Response Criteria for Intratumoral Immunotherapy in Solid Tumors.
