# Supplementary material for: Phase 1b Study of Dazostinag plus Pembrolizumab after Hypofractionated Radiotherapy in Patients with Select Advanced Solid Tumors
Source: Cancer Res Commun. 2025 Dec 31;5(12):2249–63. doi: 10.1158/2767-9764.CRC-25-0566 (PMC12754119; doi:10.1158/2767-9764.CRC-25-0566)
Supplement: Supplemental Figure S1 — Supplementary Figure S1 [file crc-25-0566_supplemental_figure_s1_suppsf1.pdf]

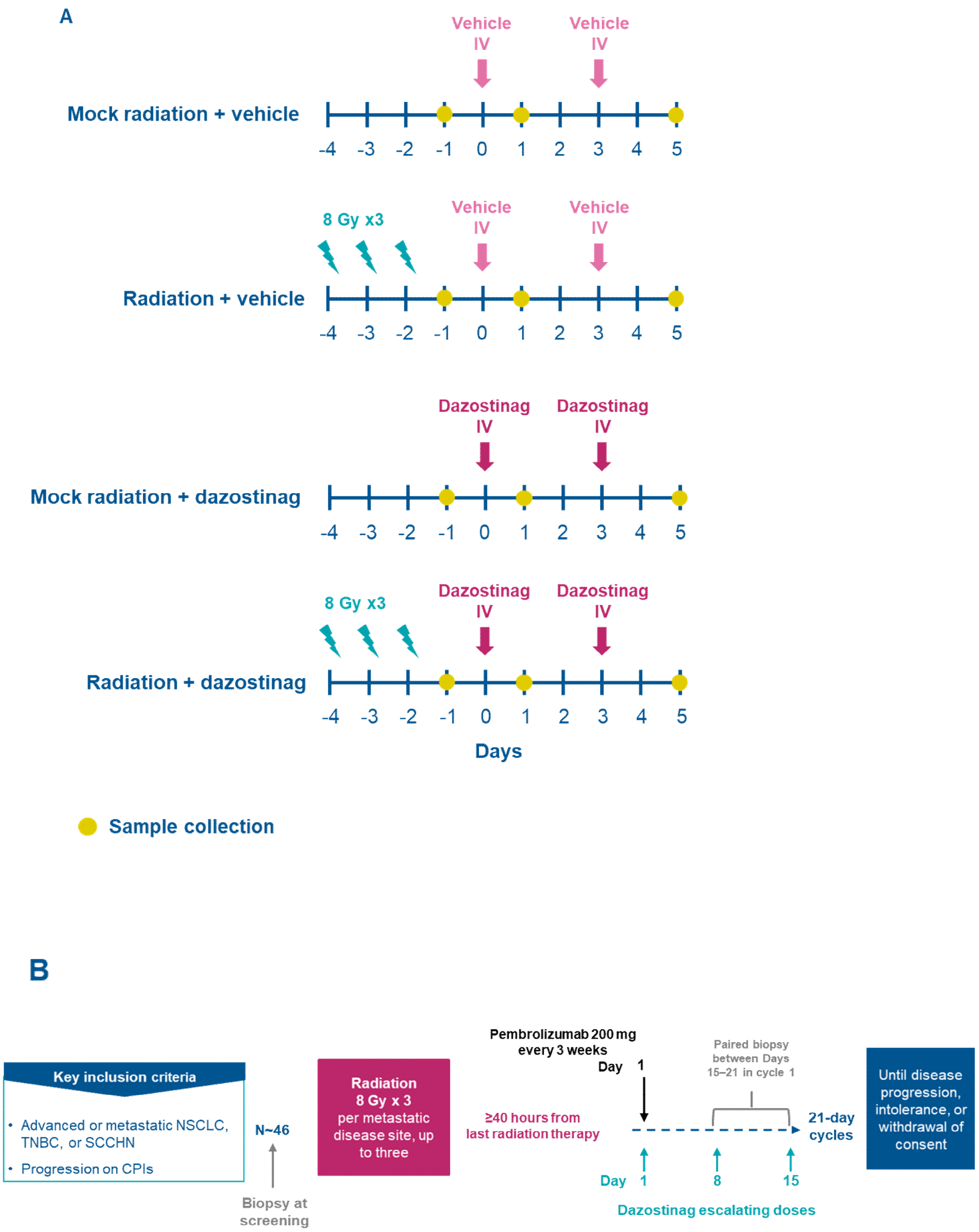

Supplemental Figure S1 (A) Preclinical (pharmacodynamic evaluation) and (B) clinical study designs. CPI, checkpoint inhibitor; Gy, Gray; IV, intravenous; IV, intravenous; NSCLC, non-small cell lung cancer; SCCHN, squamous cell carcinoma of the head and neck; TBNC, triple-negative breast cancer.
