# Supplementary material for: Phase 1b Study of Dazostinag plus Pembrolizumab after Hypofractionated Radiotherapy in Patients with Select Advanced Solid Tumors
Source: Cancer Res Commun. 2025 Dec 31;5(12):2249–63. doi: 10.1158/2767-9764.CRC-25-0566 (PMC12754119; doi:10.1158/2767-9764.CRC-25-0566)
Supplement: Supplemental Figure S2 — Supplementary Figure S2 [file crc-25-0566_supplemental_figure_s2_suppsf2.pdf]

A

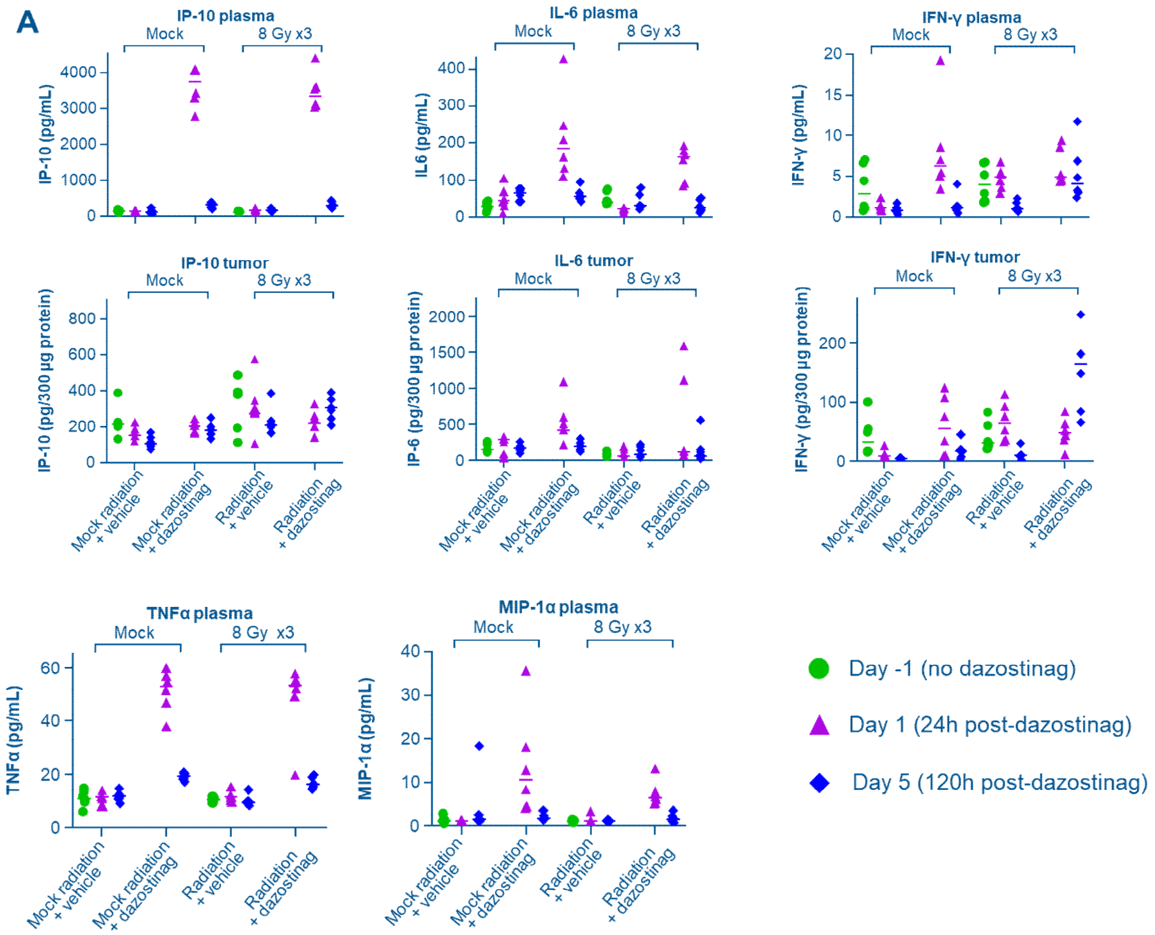
IP-10 plasma
IP-10 (pg/mL)
IL-6 plasma
IL6 (pg/mL)
IFN-γ plasma
IFN-γ (pg/mL)
IP-10 tumor
IP-10 (pg/300 µg protein)
IL-6 tumor
IP-6 (pg/300 µg protein)
IFN-γ tumor
IFN-γ (pg/300 µg protein)
TNFα plasma
TNFα (pg/mL)
MIP-1α plasma
MIP-1α (pg/mL)
Mock
8 Gy x3
Mock radiation + vehicle
Mock radiation + dazostinag
Radiation + vehicle
Radiation + dazostinag
Day -1 (no dazostinag)
Day 1 (24h post-dazostinag)
Day 5 (120h post-dazostinag)

B

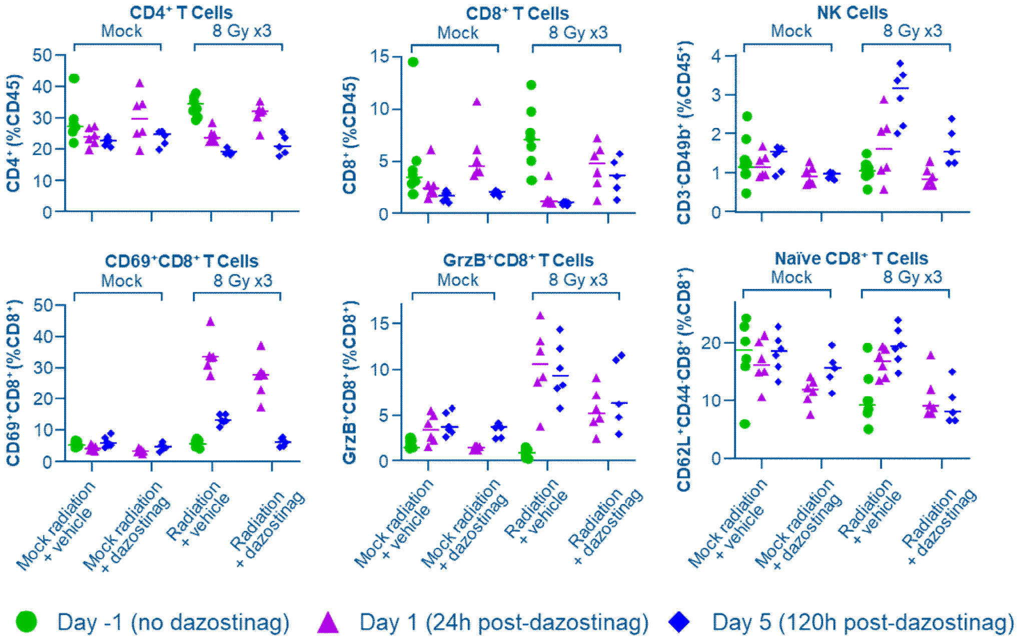
CD4⁺ T Cells
CD4⁺ (%CD45)
CD8⁺ T Cells
CD8⁺ (%CD45)
NK Cells
CD3⁻CD49b⁺ (%CD45⁺)
CD69⁺CD8⁺ T Cells
CD69⁺CD8⁺(%CD8⁺)
GrzB⁺CD8⁺ T Cells
GrzB⁺CD8⁺ (%CD8⁺)
Naïve CD8⁺ T Cells
CD62L⁺CD44⁻CD8⁺ (%CD8⁺)
Mock
8 Gy x3
Mock radiation + vehicle
Mock radiation + dazostinag
Radiation + vehicle
Radiation + dazostinag
Day -1 (no dazostinag)
Day 1 (24h post-dazostinag)
Day 5 (120h post-dazostinag)

Supplemental Figure S2 (A) Preclinical pharmacodynamic analyses of cytokine levels from plasma and tumor samples of EMT6 tumor-bearing mice following radiation and/or dazostinag treatment assessed by Meso Scale Discovery multiplex cytokine assays. (B) Immunophenotyping assays from tumor-draining lymph nodes of tumor-bearing mice treated with radiation and dazostinag assessed by flow cytometry**.** GrzB, granzyme B; Gy, Gray; IFN-γ, interferon gamma; IL6, interleukin-6; IP-10, interferon gamma-induced protein 10; MIP-1α, macrophage inflammatory protein-1 alpha; mock, mock radiation treatment; NK, natural killer; TNFα, tumor necrosis factor alpha.
