# Supplementary material for: Phase 1b Study of Dazostinag plus Pembrolizumab after Hypofractionated Radiotherapy in Patients with Select Advanced Solid Tumors
Source: Cancer Res Commun. 2025 Dec 31;5(12):2249–63. doi: 10.1158/2767-9764.CRC-25-0566 (PMC12754119; doi:10.1158/2767-9764.CRC-25-0566)
Supplement: Supplemental Figure S3 — Supplementary Figure S3 [file crc-25-0566_supplemental_figure_s3_suppsf3.pdf]

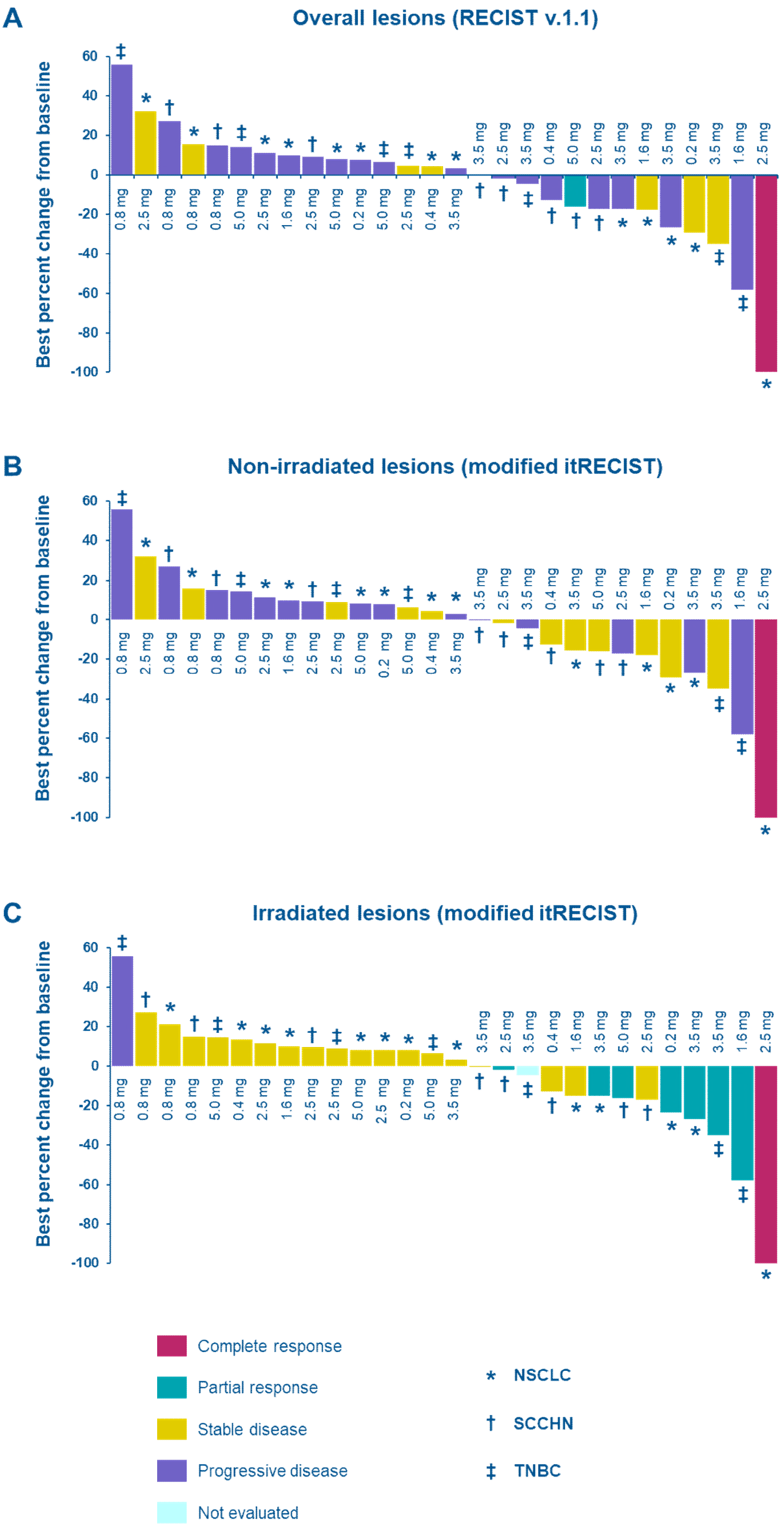
A
Overall lesions (RECIST v.1.1)
Best percent change from baseline
60
40
20
0
-20
-40
-60
-80
-100
0.8 mg
2.5 mg
0.8 mg
0.8 mg
0.8 mg
5.0 mg
2.5 mg
1.6 mg
2.5 mg
5.0 mg
0.2 mg
5.0 mg
2.5 mg
0.4 mg
3.5 mg
3.5 mg
2.5 mg
3.5 mg
0.4 mg
5.0 mg
2.5 mg
3.5 mg
1.6 mg
3.5 mg
0.2 mg
3.5 mg
1.6 mg
2.5 mg
B
Non-irradiated lesions (modified itRECIST)
Best percent change from baseline
60
40
20
0
-20
-40
-60
-80
-100
0.8 mg
2.5 mg
0.8 mg
0.8 mg
0.8 mg
5.0 mg
2.5 mg
1.6 mg
2.5 mg
2.5 mg
5.0 mg
0.2 mg
5.0 mg
0.4 mg
3.5 mg
3.5 mg
2.5 mg
3.5 mg
0.4 mg
3.5 mg
5.0 mg
2.5 mg
1.6 mg
0.2 mg
3.5 mg
3.5 mg
1.6 mg
2.5 mg
C
Irradiated lesions (modified itRECIST)
Best percent change from baseline
60
40
20
0
-20
-40
-60
-80
-100
0.8 mg
0.8 mg
0.8 mg
0.8 mg
5.0 mg
0.4 mg
2.5 mg
1.6 mg
2.5 mg
2.5 mg
5.0 mg
2.5 mg
0.2 mg
5.0 mg
3.5 mg
3.5 mg
2.5 mg
3.5 mg
0.4 mg
1.6 mg
3.5 mg
5.0 mg
2.5 mg
0.2 mg
3.5 mg
3.5 mg
1.6 mg
2.5 mg
Complete response
Partial response
Stable disease
Progressive disease
Not evaluated
* NSCLC
† SCCHN
‡ TNBC

Supplemental Figure S3 Best percentage change from baseline in sum of target lesion diameter, per RECIST v1.1 criteria (A), in sum of target non-irradiated lesion diameter per modified itRECIST criteria (B), or in sum of target irradiated lesion diameter per modified itRECIST criteria (C). CR, complete response; NE, not evaluable; NSCLC, non-small cell lung cancer; PD, progressive disease; itRECIST 1.1, intratumoral immunotherapy RECIST version 1.1.; PR, partial response; RECIST v1.1., Response Evaluation Criteria in Solid Tumors version 1.1.; SCCHN, squamous cell carcinoma of the head and neck; SD, stable disease; TNBC, triple-negative breast cancer cell;
