# Supplementary material for: Phase 1b Study of Dazostinag plus Pembrolizumab after Hypofractionated Radiotherapy in Patients with Select Advanced Solid Tumors
Source: Cancer Res Commun. 2025 Dec 31;5(12):2249–63. doi: 10.1158/2767-9764.CRC-25-0566 (PMC12754119; doi:10.1158/2767-9764.CRC-25-0566)
Supplement: Supplemental Figure S4 — Supplementary Figure S4 [file crc-25-0566_supplemental_figure_s4_suppsf4.pdf]

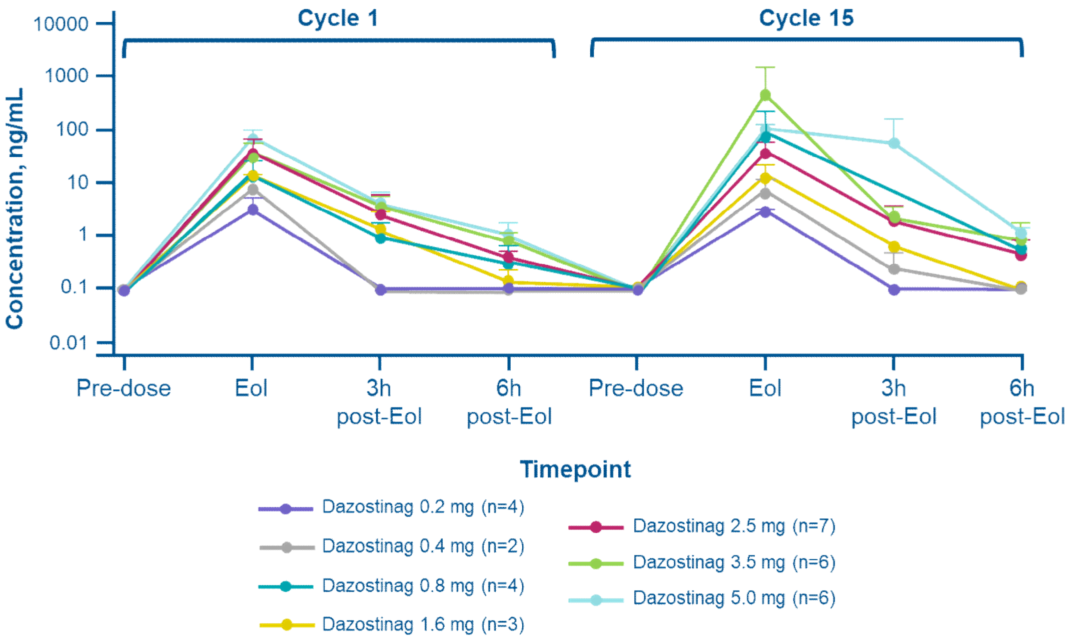

Supplemental Figure S4 Mean (standard deviation) dazostinag plasma concentration by time point and dosing cohorts**.** EoI, end of infusion.
